# Daily Brain Metabolic Rhythms of Wild Nocturnal Bats

**DOI:** 10.3390/ijms25189850

**Published:** 2024-09-12

**Authors:** Tianhui Wang, Hui Wang, Yujia Chu, Mingyue Bao, Xintong Li, Guoting Zhang, Jiang Feng

**Affiliations:** 1 College of Life Science, Jilin Agricultural University, Changchun 130118, China; wangtianhui@mails.jlau.edu.cn (T.W.); chuyujia@mails.jlau.edu.cn (Y.C.); baomingyue@mails.jlau.edu.cn (M.B.); 20232059@mails.jlau.edu.cn (X.L.); zhangguoting@mails.jlau.edu.cn (G.Z.); 2 Jilin Provincial International Cooperation Key Laboratory for Biological Control of Agricultural Pests, Changchun 130118, China; 3 Jilin Provincial Key Laboratory of Animal Resource Conservation and Utilization, Northeast Normal University, Changchun 130117, China

**Keywords:** bats, brain, circadian rhythm, metabolomics

## Abstract

Circadian rhythms are found in a wide range of organisms and have garnered significant research interest in the field of chronobiology. Under normal circadian function, metabolic regulation is temporally coordinated across tissues and behaviors within a 24 h period. Metabolites, as the closest molecular regulation to physiological phenotype, have dynamic patterns and their relationship with circadian regulation remains to be fully elucidated. In this study, untargeted brain metabolomics was employed to investigate the daily rhythms of metabolites at four time points corresponding to four typical physiological states in *Vespertilio sinensis*. Key brain metabolites and associated physiological processes active at different time points were detected, with 154 metabolites identified as rhythmic. Analyses of both metabolomics and transcriptomics revealed that several important physiological processes, including the pentose phosphate pathway and oxidative phosphorylation, play key roles in regulating rhythmic physiology, particularly in hunting and flying behaviors. This study represents the first exploration of daily metabolic dynamics in the bat brain, providing insights into the complex regulatory network of circadian rhythms in mammals at a metabolic level. These findings serve as a valuable reference for future studies on circadian rhythms in nocturnal mammals.

## 1. Introduction

The circadian rhythm is one of the biological rhythms that organisms have developed through evolution to adapt to the rotation of the earth. It manifests as a periodic rhythm regulated by the internal biological clock [1]. Important behaviors and physiological processes in mammals exhibit dynamic rhythms for over a 24 h period, including feeding, sleeping, body temperature, cell metabolism, and proliferation [2,3]. The biological clock system is organized in a hierarchical manner and is distributed within organs, tissues, and cells [4,5]. The “master clock” tissue in the brain, the suprachiasmatic nucleus (SCN), is the primary source of circadian clock signals in mammals. Its rhythmic output regulates both the auxiliary circadian oscillator in the brain and peripherally. Previous research has shown that light information input to the circadian system is transmitted through the retinohypothalamic tract (RHT) neuroglia cells to the core SCN [6]. Emerging evidence indicates that rhythmic light information is not only transmitted to the suprachiasmatic nucleus (SCN) but also directly or indirectly influences various other brain regions, thereby regulating time-dependent rhythmic brain functions throughout the day [7]. In the previous study, by exposing mice to disruption of the light-dark cycle from birth until weaning, researchers found that this early-life circadian disruption may have long-lasting and far-reaching effects on their behavior and brain development [8]. Several studies have shown that there were strong associations between disrupted circadian rhythms and disorders of the central nervous system, resulting in a high risk of brain diseases in humans [9,10]. Moreover, in addition to SCN, a number of areas of the mammalian brain showed circadian rhythms in core clock gene expression, hormone output, and nervous activity [11]. Therefore, gaining insights into the circadian rhythms of the brain is of great necessity and is important for the understanding of regulatory mechanisms underlying periodic adaptation to the environment of mammals and also circadian rhythm-related conditions in humans.

Recently, the molecular regulatory network of circadian rhythms in the brain has been clearly revealed. At the molecular level, circadian rhythms are generated by an integrated network of clock genes and proteins that interact in a transcription–translation feedback loop (TTFL) [12]. It is primarily the protein complex encoded by the *Clock* and *BMAL1* genes that activates the transcriptional translation feedback loop composed of the *Per* and *Cry* genes, rhythmically regulated to maintain the approximately 24 h cycle in the brain and body cells. Furthermore, current research has demonstrated the existence of metabolic oscillators and the metabolic state of cells could directly regulate the rhythmic genes and transcriptional control [13]. Metabolites are the intermediate products or the end result of transcriptional activity and protein expression, involving the release of cell signals, energy transfer, and intercellular communication, as well as being the material carriers of many life activities and indicators of physiological states. Changes in metabolic levels are thought to be the ultimate response of biological systems to genetic or environmental changes. Some metabolites, such as cAMP [14], melatonin, and a variety of neurotransmitters that could affect the expression of circadian clock genes [15], are considered to be closely related to the regulation of circadian rhythms. A number of researches indicate that melatonin is essential for controlling sleep–wake cycles and circadian rhythm [16,17]. It is a hormone produced by the pineal gland in the brain, characterized by a marked increase in secretion during the dark phase, and serves as an important neuroendocrine output of the biological clock [18]. Moreover, both diurnal humans and nocturnal rats experience an increase in melatonin levels during their own dark periods. Moreover, metabolomics has been proven to be useful in studies about circadian clock function in the liver and blood of mice and also has been applied to blood samples of humans to investigate the effect of sleep deprivation [19]. Therefore, the molecular mechanisms underlying circadian rhythms represent a complex system that involves gene transcriptional regulation, feedback regulation, post-translational modification, as well as metabolite and environmental signaling regulation, among other levels. These processes may work in concert to maintain the rhythms of organisms across varying physiological states and environmental conditions. With the development and application of molecular biology techniques, there is an opportunity to jointly elucidate the complex regulatory network of mammalian circadian rhythms at a multi-molecular level, revealing important rhythmic molecules and the physiological processes they influence or regulate can help us to gain a clearer understanding of mammalian circadian rhythms. At the same time, it may provide deeper insights into the conservation and diversity of mammalian metabolic rhythms.

The temporal niche an animal occupies influences a coordinated set of behavioral and physiological processes that distinguish diurnal animals from nocturnal animals. At present, research on circadian rhythms is focused on model animals under controlled laboratory conditions, such as mice [20]. Although mice are nocturnal, they are often subjected to artificial time adjustments in the laboratory, which may differ from the rhythmic regulation mechanisms of nocturnal animals in natural habitats. Bats belong to Chiroptera, depending on developed echolocation ability and flight ability [21], bats become strictly nocturnal mammals to adapt to the night sky life. Bats have evolved a high degree of species diversity, and are the second largest group of mammals in terms of species number, second only to rodents [22]. Although previous studies have reported the daily transcriptomes of the brain and liver, along with associated physiological processes in *Vespertilio sinensis* (Asian particolored bat), our understanding of bat circadian rhythms at the molecular level remains limited. In addition, studying the regulatory mechanism underlying circadian rhythm in bats from the perspective of a single transcriptome may be one-sided for the multifaceted regulatory mechanism of circadian rhythm, and it is necessary to deeply integrate the action processes and mechanisms of macromolecules, like metabolisms, that are closely related with physiological phenotype.

In this study, we conducted comparative metabolomics to identify and compare, for the first time, metabolites in the brain of *V. sinensis* under four successive time points corresponding to four biological states across 24 h. The four time points were set as the same as the previous study with the daily brain transcriptomes of Asian particolored bats [23], aiming to identify key rhythmic metabolites and related physiological processes in the brain. Combined with the bat behavior state, we aimed to reveal the significance of rhythmic metabolites and processes in the regulation of circadian rhythm. Concurrently, integrated with transcriptomic data, gene and metabolite analyses were conducted to elucidate the molecular regulatory mechanisms of circadian rhythms in the bat brain from a multimolecular viewpoint. The results of this study will systematically clarify the rhythmic metabolic bases in the brain of nocturnal bats, deepen our understanding of the molecular and physiological mechanisms underlying circadian rhythm regulation in Chiroptera, and provide new data reference and research ideas for other non-model organisms related to circadian rhythms.

## 2. Results

### 2.1. Classification of Bat Brain Metabolites

**Figure 1:**
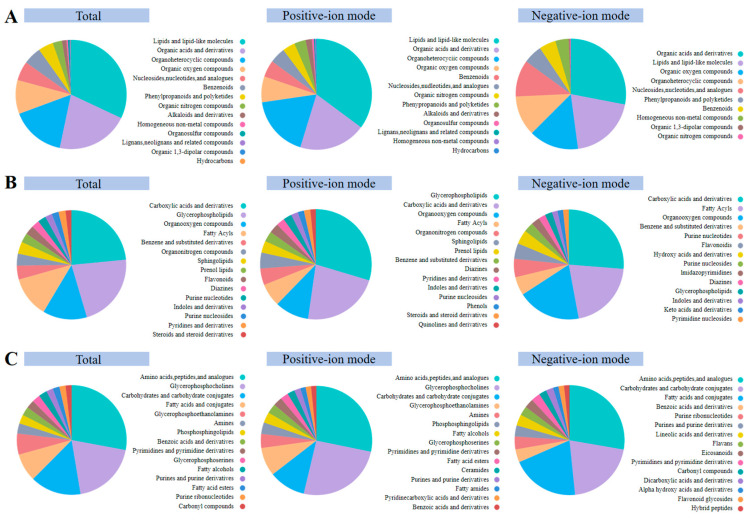
Annotated metabolites in the bat brain were detected for (**A**) super-classification, (**B**) classification, and (**C**) sub-classification, respectively.

A total of 903 annotated metabolites were detected in the brain of Asian particolored bat by LC-MS (Table S1), and there were 892, 871, 673, 495, 259, and 645 metabolites annotated by the HMDB, PubChem, CAS, KEGG, METLIN, and ChEBI databases, respectively (Table S2). The metabolites were further divided into 14 categories at the super-class level, with lipids and lipid-like molecules, organic acids and derivatives, organoheterocyclic compounds, organic oxygen compounds, and nucleosides, nucleotides, and analogs being the top five largest metabolic categories (Figure 1A and Table S3). The distribution of metabolites at the Class and Sub-class levels is presented in Figure 1B and 1C, respectively.

**Figure 2:**
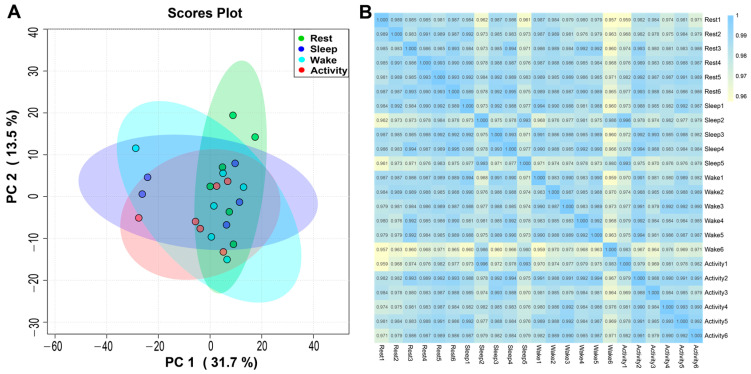
Results of inter-sample relationships. (**A**) Metabolite principal component analysis (PCA) among samples of the brain at different states. (**B**) The heat map of sample correlation analysis.

Preliminary PCA results indicated that sleep 6 was a typical outlier sample (Figures S1 and S2). The PCA diagram with the deletion of sleep 6 showed good repeatability between samples from the same group (Figure 2A), similar results were obtained by inter-sample correlation analysis (Figure 2B). Therefore, all following analyses were performed using 23 samples from four physiological states without sleep 6. In addition, OPLS-DA results showed a clear separation of samples from each time point corresponding to the four physiological states (Figures S3 and S4).

### 2.2. DAMs Detected between Different Physiological States

**Figure 3:**
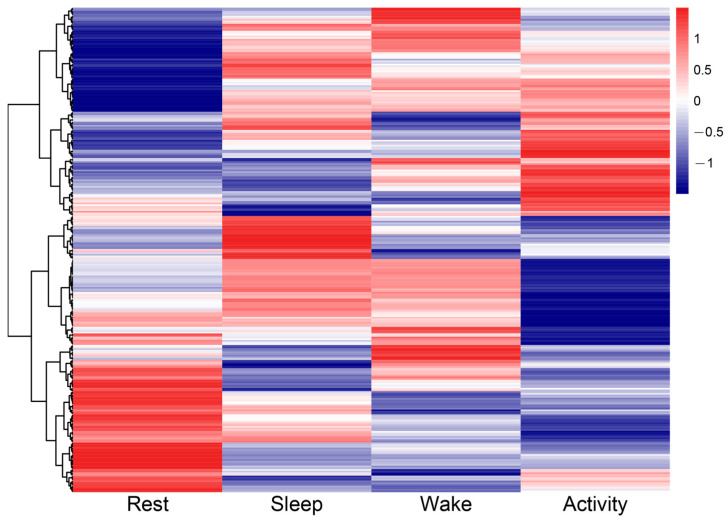
Heatmaps of 293 DAMs from six pairwise comparisons across four time points corresponding to four physiological states. The four columns represent the amount of each DAM detected in the rest, sleep, wake, and activity state, respectively.

A total of 639 DAMs were detected in the six pairwise comparisons (Table 1 and Table S4). In the activity vs. rest comparison, the number of DAMs was the highest, with 165 metabolites (96 and 69 metabolites were significantly abundant in the rest and active state, respectively). The lowest number of DAMs was detected in the sleep vs. wake comparison, with 48 metabolites (28 and 20 metabolites were significantly abundant in the sleep and wake state, respectively). Notably, a number of DAMs were detected in two or more pairwise comparisons and, after removing repeatedly detected DAMs, there were 293 out of 639 DAMs remaining for downstream analyses. A heatmap of the hierarchical clustering of 293 DAMs showed obvious differences in the abundance of metabolites between different time points corresponding to four physiological states (Figure 3).

**Figure 4:**
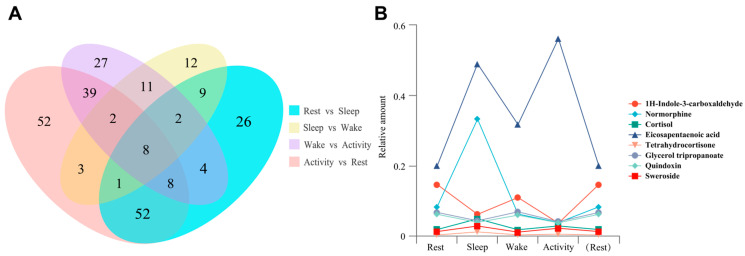
(**A**) Venn diagram of DAMs from four pairwise comparisons involved every two adjacent time points. (**B**) The change patterns of eight common metabolites from four pairwise comparisons detected. Raw data normalization was conducted using MetaboAnalystR 4.0 [24], which was integrated within the R software version 4.2.3 by utilizing the sum of features for each sample.

The Venn diagram shows that there were eight common DAMs shared by four comparisons involved every two adjacent time points. Namely, the eight metabolites, including 1H-Indole-3-carboxaldehyde, Normorphine, Cortisol, Eicosapentaenoic acid, Tetrahydrocortisone, Glycerol tripropanoate, Quindoxin, and Sweroside, accumulated significantly different between every two adjacent time points (Figure 4A). A visualization of these eight metabolites across four time points is shown in Figure 4B.

Moreover, analysis of DAMs from rest vs. wake comparison involving two opposite states indicated that t 42 metabolites were significantly accumulated in the rest state (after hunting) and 62 metabolites were significantly accumulated in the wake state (before hunting). For the sleep vs. activity comparison, there were 79 and 32 metabolites significantly accumulated in the sleep and active state, respectively.

### 2.3. KEGG Pathways Enriched by DAMs

**Figure 5:**
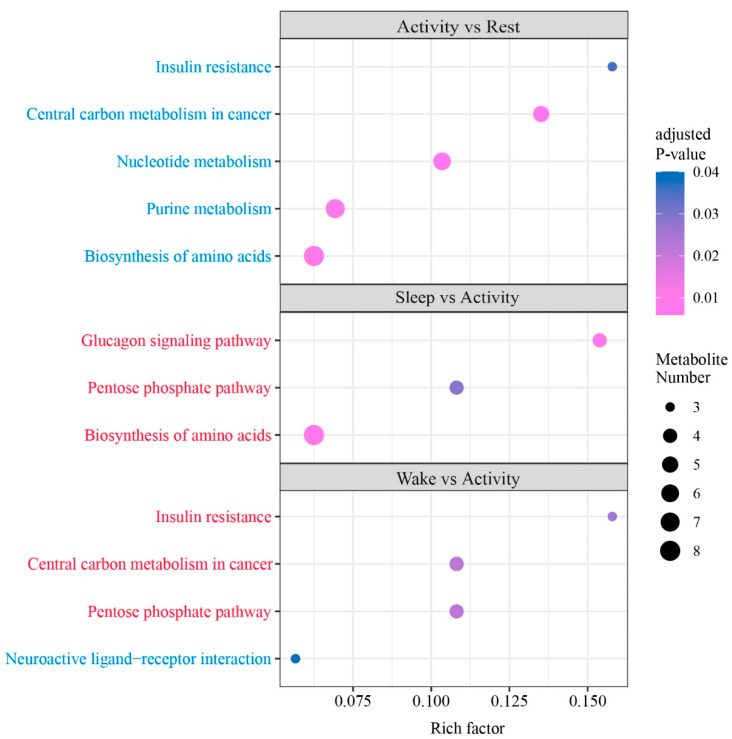
KEGG pathways were significantly enriched by DAMs detected from six pairwise comparisons. Pathways enriched by higher abundant metabolites detected in the former state than in the latter of one comparison are shown in red, and pathways enriched by higher abundant metabolites detected in the latter state are shown in blue. Significant pathways were obtained by KEGG enrichment analysis of DAMs in six pairwise comparison groups. Pathways significantly enriched in DAMs that were more abundant in the previous state in the comparison group are shown in red. Pathways significantly enriched in DAMs that were richer in the latter state in the comparison group are shown in blue.

DAMs from three of six comparisons were significantly enriched in various KEGG pathways (Figure 5 and Table 1). Specifically, in the activity vs. rest comparison, DAMs that were more abundant in the rest state were significantly enriched in pathways of Nucleotide metabolism (ko01232), Biosynthesis of amino acids (ko01230), Purine metabolism (ko00230), and Insulin resistance (ko04931). Sleep and activity were the two opposite states for the day and night of bats, in the sleep vs. activity comparison, DAMs that were more abundant in the sleep compared with the active state were significantly enriched in the Biosynthesis of amino acids (ko01230), Glucagon signaling pathway (ko04922), and Pentose phosphate pathway (ko00030) pathways. DAMs that were more abundant in the wake state compared with the active state were significantly enriched in the Pentose phosphate pathway (ko00030), Central carbon metabolism in cancer (ko05230), and Insulin resistance (ko04931), whereas DAMs that were more abundant in the active state compared with wake state were significantly enriched in the Neuroactive ligand–receptor interaction (ko04080). DAMs from rest vs. sleep, sleep vs. wake, and rest vs. wake comparisons did not significantly enrich any of the pathways (Table S5).

**Figure 6:**
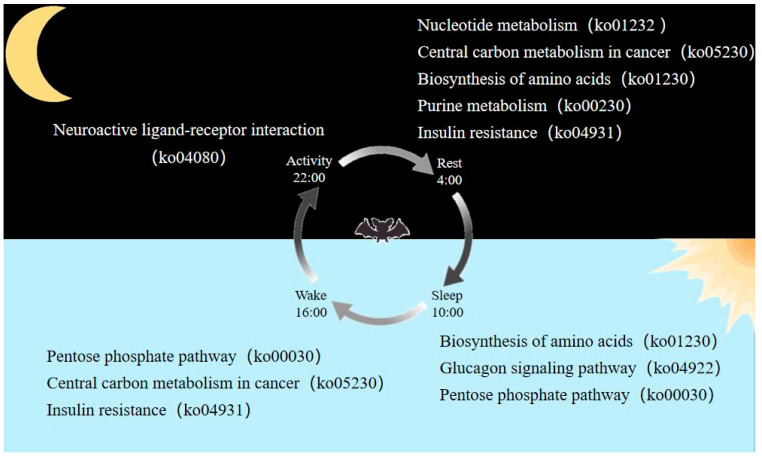
Summary of the more active physiological processes for each time point in the bat brain.

Important physiological processes underlying the significant enrichment of abundant DAMs at each time point are summarized in Figure 6. It indicated that metabolites with high abundance in rest and wake participated in the same process of Insulin resistance, and the high abundance metabolites detected in sleep were involved in the Glucagon signaling pathway. Together, these findings suggest that both physiological processes, which are crucial for the regulation of blood sugar balance, exhibited dynamic changes throughout the day.

In the comparisons of sleep vs. activity and wake vs. activity, metabolites with high abundance in sleep and wake were significantly enriched in the physiological process of the Pentose phosphate pathway. This indicated that this pathway was more active during both sleep and wake. The Pentose phosphate pathway primarily involves the conversion of glucose-6P from glycolysis to ribulose-5P through oxidative decarboxylation, and then to ribose-5-phosphate. In the comparisons of activity vs. rest and sleep vs. activity, metabolites with high abundance in rest and sleep were significantly enriched in the Biosynthesis of amino acids. This finding suggested that the process of Biosynthesis of amino acids is more active during rest and sleep in the brains of Asian particolored bats.

### 2.4. Clusters of Metabolites and Associated Pathways across Four Time Points

**Figure 7:**
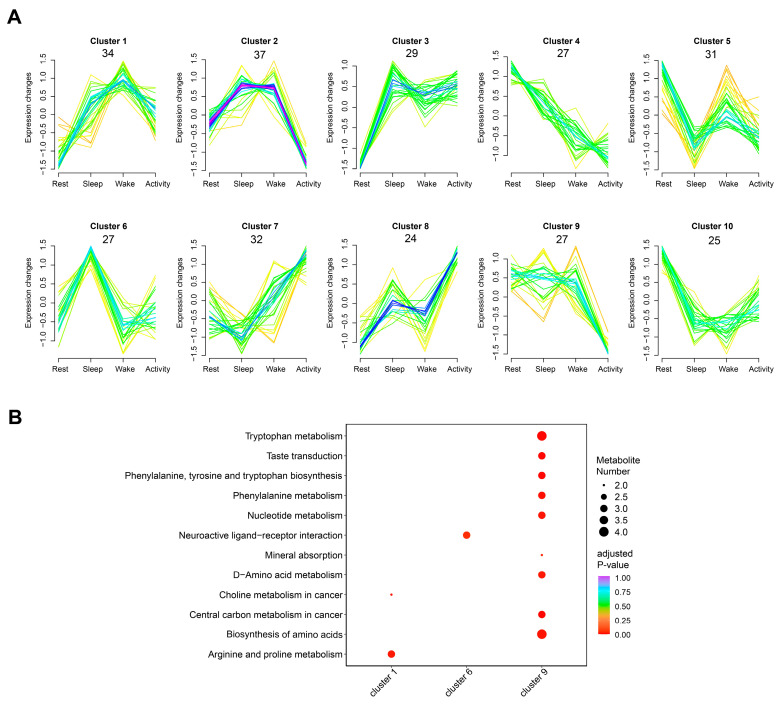
Clusters of 293 DAMs according to their change patterns across four time points. (**A**) Ten clusters of DAMs. The number under the cluster indicates the DAMs clustered in it. (**B**) KEGG pathways were significantly enriched by DAMs from cluster 1, cluster 6, and cluster 9 (DAMs from other clusters were not significantly enriched in any pathway).

To clarify the accumulated patterns of metabolites in the brain of Asian particolored bats during a 24 h period, we examined the union set of the six groups of DAMs, and a total of 293 metabolites were obtained. All these 293 DAMs from six comparisons were classified into 10 clusters (Figure 7A and Table S6). Particularly, metabolites such as melatonin, cortisol, and adenosine, which were highly correlated with circadian rhythmic activities, were found in cluster 6. With abundance gradually increased from rest to sleep state and peaked in the sleep state, then gradually decreased until the wake state, suggesting related physiological processes in which these metabolites involved may also exhibit similar patterns. Downstream enrichment analysis indicated that the metabolites from cluster 6 were significantly enriched in the Neuroactive ligand-receptor interaction pathway (ko04080) (Figure 7B).

### 2.5. Rhythmic Metabolites in the Brain of Asian Particolored Bat

**Figure 8:**
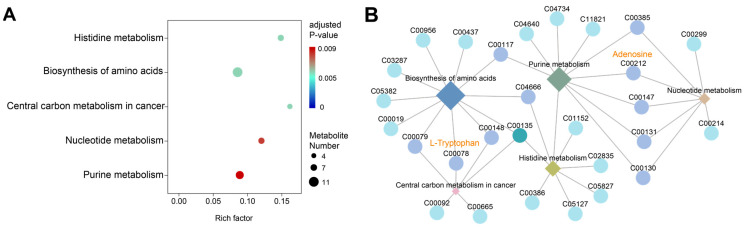
(**A**)The KEGG pathways were significantly enriched by rhythmic DAMs. (**B**) The KEGG pathway–metabolite interaction network analysis. Diamonds represent pathways, circles represent metabolites.

To better uncover the rhythmic metabolites in the brain of Asian particolored bats, rhythmic analyses were performed for all metabolites and the DAMs, respectively. There were 214 rhythmic metabolites identified from all metabolites, with a rhythmic proportion of 23.7% (214/903 = 0.23). Meanwhile, there were 154 rhythmic metabolites out of 293 DAMs detected by both algorithms (Table S7), with a rhythmic proportion of 52.6% (154/293 = 0.52). Rhythmic DAMs were considered to be more significant for downstream analyses. Figure 8A showed pathways that were significantly enriched by 154 rhythmic DAMs, including Biosynthesis of amino acids (ko01230), Nucleotide metabolism (ko01232), and Purine metabolism (ko00230), indicating that these physiological processes exhibited rhythmic changes in the bat brain. Moreover, we found that adenosine and L-tryptophan, which were circadian rhythm-related metabolites, exhibited higher connectivity in the functional-metabolite network interaction diagram (Figure 8B).

**Figure 9:**
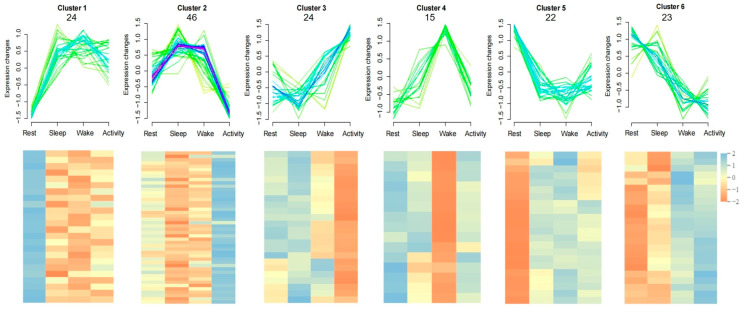
Clusters of 154 rhythmic DAMs according to their change patterns across four time points. Six clusters of rhythmic DAMs (**up**) and related heatmaps (**down**). The number under the cluster indicates the rhythmic metabolites clustered in it.

In addition, these 154 rhythmic DAMs were classified into six clusters, as shown by Figure 9, along with their abundance changes across four time points, including 24, 46, 24, 15, 22, and 23 metabolites, respectively. Metabolites in each cluster varied with time.

**Figure 10:**
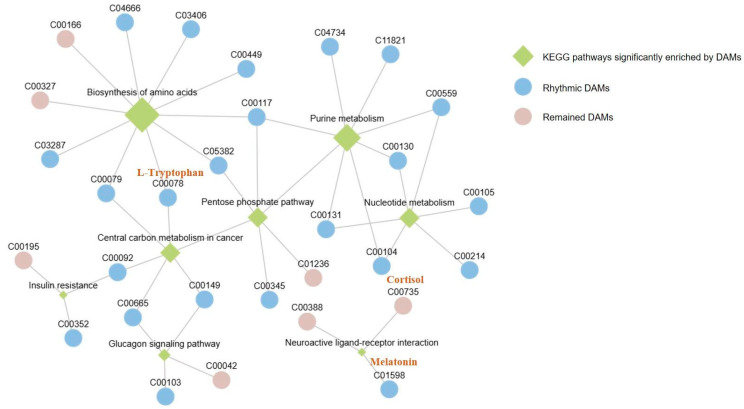
The pathway–metabolite interaction network of significantly enriched pathways from six pairwise comparisons and associated involved metabolites. The rhythmic and differential characteristics of metabolites were labeled with different colors.

**Figure 11:**
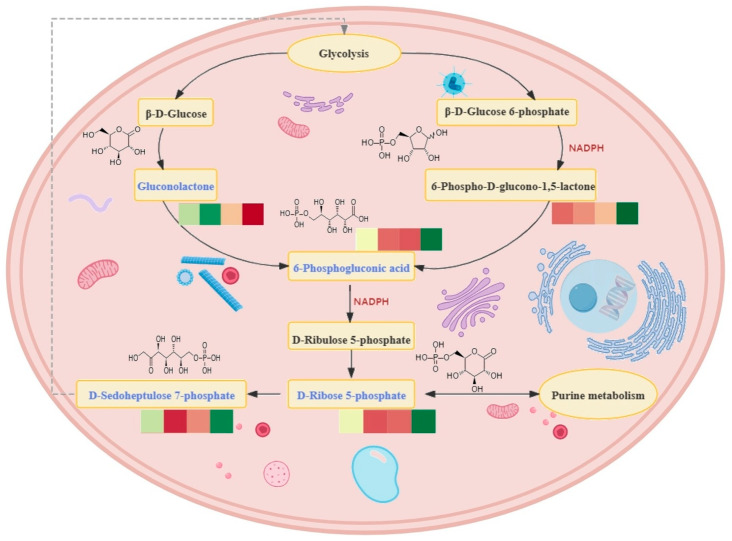
Illustration of the main process of the pentose phosphate pathway. The simple heatmap showed the content of corresponding metabolites across four time points from 4:00 to 22:00. The deeper the red color, the higher the content; the deeper the green color, the lower the content. The blue pathways showed the rhythmic metabolites detected in the brain of a bat.

Further, network relationships were conducted for the DAMs and associated significantly enriched pathways from six pairwise comparisons. After mapping the rhythmicity to the DAMs (Figure 10), we found that most of the DAMs were rhythmic. Several well-known rhythmic-regulating hormones, like L-Tryptophan, cortisol, and melatonin, were identified to be crucial rhythmic DAMs and also showed close relationships with important processes in this network. The pathways were significantly enriched by rhythmic DAMs, suggesting their similar rhythmic functions, including the Pentose phosphate pathway. In consideration of the functional importance of the Pentose phosphate pathway, five DAMs detected in this study were mapped in its pathway map, indicating that four out of five DAMs (75%) were rhythmic and were also the key materials participating in this rhythmic process (Figure 11).

### 2.6. Key Metabolites in the Brains of Bats in a Rest State

**Figure 12:**
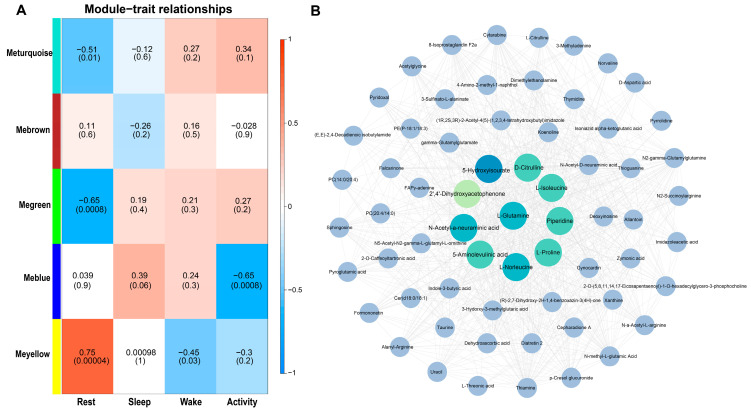
Correlation of brain metabolites with four states based on WGCNA. (**A**) Module–state relationship. (**B**) Yellow module metabolite network for the metabolites from the yellow module. The top 10 metabolites with the highest connectivity are shown with highlighted colors.

The results of WGCNA identified key metabolites for every physiological state. All metabolites were divided into five modules according to their abundance at each time point (Figure 12A and Table S8). Only the yellow module was detected to be significantly associated with the rest state (*p* < 0.05). A total of 68 metabolites were included in the yellow module and they were significantly enriched in the ABC transporters (ko02010). According to the results of the metabolite interaction network (Figure 12B), the top 10 metabolites with the highest connectivity are 5-Hydroxyisourate, L-Glutamine, N-Acetyl-a-neuraminic acid, L-Norleucine, Piperidine, D-Citrulline, L-Isoleucine, 5-Aminolevulinic acid, L-Proline, and 2′,4′-Dihydroxyacetophenone, which have been identified as key metabolites for the rest state.

### 2.7. Results of Correlation Analysis between Metabolomics and Transcriptomics

Pathways significantly enriched by DEGs and DAMs from six pairwise comparisons were correspondingly compared. Among these, only the Parkinson’s disease (ko05012) (*p*-value < 0.05) was consistently identified as significant in both omics analyses for the wake vs. activity comparison. Additionally, compared with the activity state, DEGs with higher expression levels in the wake state were significantly enriched in Oxidative phosphorylation (ko00190), and accumulated DAMs in the wake state were significantly enriched in the Pentose phosphate pathway (ko00030). Both pathways were crucial in energy metabolism. To explore relationships between genes and metabolites in the bat brain, correlation analyses of DEGs and DAMs were conducted for each pairwise comparison (Table S9). Metabolites and genes with Pearson correlation coefficients greater than 0.8 were plotted in nine-quadrant diagrams (Figure S5).

**Figure 13:**
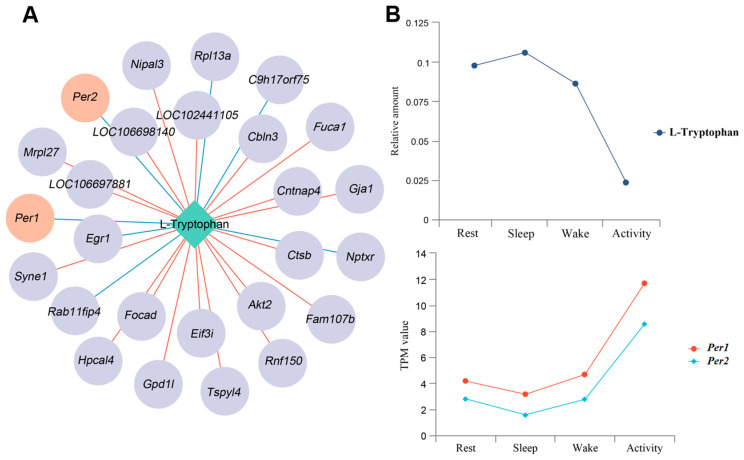
(**A**) A network of DEGs correlated with tryptophan. The red connecting line indicates a positive correlation and the blue connecting line indicates a negative correlation. (**B**) The expressed trends of tryptophan and *Per1*/*2* across four states.

Through the correlation analysis of genes and metabolites, we found that several genes were highly correlated with rhythmic L-Tryptophan across four time points (Figure 13A). In particular, there was a negative correlation between the expression levels of two essential circadian clock genes, *Per1* and *Per2*, and the amount of L-tryptophan across time (Figure 13B).

## 3. Discussion

Mammals rely on the circadian clock network to regulate daily systemic metabolism and physiological activities, including hormone release, heartbeat, homeostasis maintenance, and sleep–wake cycle [25]. As totally wild nocturnal mammals, bats are more enigmatic and are known for their abilities to fly and echolocate in the dark; however, little is known about the molecular bases underlying daily rhythms. In recent years, bats have emerged as new models for the neurobiology of navigation, social neuroscience, aging, and immunity [26]. Bats exhibit higher metabolic rates and utilize twice as much energy over their lifetimes compared to other mammals [27]. This unique metabolic adaptation makes them a new animal model for studying metabolic rhythms. It is anticipated that bats, with high metabolic levels, can contribute to novel strategies for addressing human brain metabolism-related disorders in the future. Moreover, given bats’ longevity among small mammals [28], research into their brain metabolism could also hold promise for aging studies.

For the first time, this study revealed the rhythmic metabolites and associated physiological processes in the brain of nocturnal bat species. We found that 23.7% of all metabolites exhibited rhythmic, 52.6% of DAMs were rhythmic, and 75% of DAMs that were significantly enriched in specific pathways were rhythmic, indicating that differentially accumulated metabolites over time, especially those enriched in important metabolic processes, could be more rhythmical. Furthermore, metabolic processes, such as the pentose phosphate pathway, may play important roles in the rhythmic behaviors of bats. By integrating bat brain transcriptome data from four similar physiological states, relationships between genes and metabolites were revealed, thereby deciphering the rhythmic energy metabolism process and potential relationships between specific metabolites and the core circadian clock gene. These findings on metabolic rhythms in bat brains enhance our understanding of animal chronobiology and biodiversity.

Various metabolites were detected in the bat brain and the five most abundant categories were Lipids and lipid-like molecules, Organic acids and derivatives, Organoheterocyclic compounds, Organic oxygen compounds, and Nucleosides, nucleotides, and analogs. Among the various metabolites detected in the bat brain, Lipids and lipid-like molecules constituted the largest proportion due to the high endogenous content of brain lipids, which form a significant structural component of the brain and are rich sources of metabolic energy [29]. Moreover, bioactive lipids play a crucial role in signaling and regulation, facilitating neurogenesis, synaptogenesis, and intercellular communication [30]. Following Lipids and lipid-like molecules are Organic acids and derivatives, including fatty acids, etc. Organic acids participate in the tricarboxylic acid cycle and oxidation reactions, playing key roles in energy synthesis and gluconeogenesis [31,32]. These metabolites, along with Organoheterocyclic compounds, Organic oxygen compounds, Nucleosides, nucleotides, and analogs, collectively maintain basic brain physiological functions and activities. These metabolic categories were similar to those obtained in previous studies on the brains of rats [33]. However, the only slight discrepancy is a higher proportion of Organoheterocyclic compounds found in the brains of bats compared with those of rats. Both bats and rats are nocturnal mammals, thus possessing conservative functions and similar metabolites in the brain to maintain normal life activities. However, the different behavioral characteristics and biological features of bats and rats may result in slightly different metabolic abundances. Bats have evolved powerful flight and echolocation abilities to adapt to nocturnal activities, which suggests high levels of neural activity and metabolism. Brain metabolism is essential for supporting complex neural signaling processes requiring many neurotransmitters, such as dopamine, glutamate, and gamma-aminobutyric acid (GABA). Organoheterocyclic compounds may play an important role in neurotransmission through interactions with neurotransmitters, hormones, or receptors [34,35]. Therefore, bats’ metabolic pathways may be more inclined toward the production of Organoheterocyclic compounds, reflecting differences in metabolite abundance in the brain due to evolutionary backgrounds, behavioral habits, dietary variations, and other environmental factors among animals. Nonetheless, the qualitative indicators of overall metabolites are similar, warranting further exploration in future studies.

### 3.1. Active Pentose Phosphate Pathway before Flight Activity

Compared to the active state, the higher abundance of metabolites detected in the sleep and wake states before hunting were significantly enriched in the pentose phosphate pathway, suggesting a more active pentose phosphate process in the brain of the Asian particolored bat before flying for hunting. Previous studies have demonstrated that the pentose phosphate pathway in the cytoplasm primarily supplies ribose for nucleotide synthesis and generates a substantial amount of NADPH [36]. Pharmacological studies have shown that the pentose phosphate pathway extends the circadian cycle in human cells, mouse tissues, and fruit flies and is a key regulator of the reoxidation and transcriptional oscillations of circadian rhythms, mainly controlled through NADPH metabolism [37]. NADPH not only serves as a reducing coenzyme but is also crucial for the synthesis of fatty acids, sterols, nucleotides, and non-essential amino acids [38,39], participating in a variety of redox reactions within cells. Furthermore, NADPH is a vital component of antioxidant defense, acting as a substrate for NADPH oxidase (NOXs), which generates reactive oxygen species (ROS) [40], aiding in the clearance of free radicals and peroxides and protecting cells from oxidative stress. Thus, the process of generating NADPH via the PPP to combat peroxidation occurs in nearly all animals. In bats, both flight and echolocation are energy-intensive and highly aerobic activities which may lead to the accumulation of peroxides in their bodies. Therefore, antioxidant mechanisms are essential to counteract oxidative stress. The highly active pentose phosphate pathway, capable of producing large quantities of NADPH before flight, may represent an adaptive physiological mechanism in bats to combat oxidative stress.

Additionally, the reduction of oxidized glutathione is also required via NADPH [41] to reduce the effects of intracellular oxidative stress on the erythrocyte membrane, which may protect the integrity of the erythrocyte membrane. One of the primary functions of red blood cells in mammals is oxygen transport. Bats require significant amounts of oxygen to support their physiological functions during flight, making it essential to maintain the morphology and functionality of red blood cells. The NADPH generated by the pentose phosphate pathway could help to protect the integrity of red blood cells in bats during flight for hunting, thereby sustaining the normal activities of bats.

Furthermore, gene–metabolite combined analyses revealed that, during the wake state, two critical physiological processes related to energy metabolism—the pentose phosphate pathway and oxidative phosphorylation—were significantly enriched by highly expressed genes and more abundant metabolites, respectively. The intermediate products of the pentose phosphate pathway contribute to glycolysis and subsequently to the citric acid cycle, resulting in the production of NADH. This reduced coenzyme serves as a vital electron donor in the oxidative phosphorylation process [42], establishing an indirect connection with the oxidative phosphorylation process. A special characteristic of bats is the ability to engage in powered flight [43]. In addition to important factors such as wing development, bats also require a higher metabolic rate, far surpassing the maximum capacity during activities of other similarly sized terrestrial mammals [44]. The oxidative phosphorylation process in mitochondria generates a substantial amount of ATP energy, elevating the metabolic level and playing a crucial role for bats. Research on the mitochondrial and nuclear genomes of bats indicates that the oxidative phosphorylation pathway has adaptively evolved to meet the demands of constantly changing environmental and physiological conditions [45]. The byproduct of oxidative phosphorylation is ROS, and excessive ROS can lead to oxidative stress. Due to its high chemical reactivity, uncontrolled ROS production will cause oxidative damage to DNA, lipids, and proteins. This oxidative stress damages the function of biomacromolecules, adversely affecting cell physiology, leading to cell damage, and ultimately inducing cell death [46]. In order to combat ROS damage, genes related to DNA damage repair in bats were found to have adaptively evolved and are under positive selection to enhance cell resistance to oxidative damage [47]. Furthermore, as an important process, the pentose phosphate pathway has also been identified in this study, which is a pathway that will produce large amounts of NADPH, reduce peroxides, and eliminate peroxidative damage induced by high metabolic levels. Overall, bats have co-evolved adaptively at the molecular level, both genetically and in terms of metabolites, to maintain a normal state of the organism. These two processes work in tandem to sustain high-energy metabolism within bats.

### 3.2. Insulin Resistance and Glucagon Keep Blood Glucose Balance in Bat Brain

Insulin and glucagon work in a coordinated manner to regulate metabolic homeostasis under various physiological conditions, such as postprandial states, fasting, and exercise [48]. Their interplay plays a critical role in blood glucose regulation, collectively influencing blood glucose levels. Glucagon is released by the alpha cells of the pancreas, can activate glucagon receptors, and is distributed in multiple tissues. Specifically, the activation of the glucagon receptor enhances the cyclic adenosine monophosphate (cAMP)–protein kinase A (PKA) signaling pathway, which promotes hepatic glucose production [49,50]. Insulin modulates the metabolic pathways that break down plasma metabolites like fatty acids, glucose, and amino acids to elevate their levels to meet the physiological demands of the body under corresponding conditions [51]. Insulin resistance occurs when cells exhibit diminished responsiveness to insulin, leading to impaired insulin sensitivity due to a reduced response of insulin signaling to blood glucose levels, resulting in a diminished physiological effect of insulin in the body [52]. This condition can be viewed as a compensatory mechanism by the body in response to energy excess. Our study found that insulin resistance was more pronounced in the brain regions of the Asian particolored bat during the rest and wake states compared to the active state, while the glucagon signaling pathway was more active during the sleep state. Generally, it could be reasonably inferred that, aside from the active state associated with hunting and feeding, the organism predominantly relies on the physiological processes of insulin resistance and glucagon signaling pathways to maintain blood glucose levels throughout the rest of the day.

### 3.3. Key Physiological Processes in Predation-Related States

Diet often influences food consumption patterns, closely linking feeding/fasting rhythms to energy metabolism rhythms [53,54]. Maintaining a regular feeding schedule may be an important factor in improving metabolic efficiency and weight stability [55]. Research on animals subjected to restricted feeding (RF) has demonstrated that RF could alter clock gene expression not only in the digestive organs but also in the cerebral cortex and hippocampus [56]. The feeding rhythm may impact the central energy metabolism regulation mechanism [57]. Current research highlights the role of diet as a critical synchronizing factor of the circadian system, particularly for peripheral tissue clocks [58]. It has been reported that the timing of food intake plays a crucial role in maintaining metabolic health or treating impaired metabolic processes in pathological conditions [59,60,61]. In the rest state, processes like nucleotide metabolism, the biosynthesis of amino acids, and purine metabolism are highly active in the bat brain [62]. Nucleotide metabolism involves the breakdown of nucleotides into ribose and bases, providing energy or raw materials needed for synthesizing other biomolecules. Meanwhile, purine metabolism is responsible for breaking down purines into uric acid, which is eventually excreted from the body. Bats typically hunt for food while active and enter a satiated state during rest. During the rest period, various material metabolic processes become active, including nucleotide metabolism and purine metabolism, which are crucial for maintaining nucleic acid synthesis and repair. In addition, a group of metabolites were significantly correlated with the rest state and were involved in the ABC transporters pathway, an important process for material transformation.

Abundant brain metabolites detected in the active state were significantly enriched in neuroactive ligand–receptor interactions, notably including melatonin and cortisol. These two hormones are rhythmic hormones closely associated with circadian rhythms [25]. The pathway of neuroactive ligand–receptor interaction is composed of various signaling molecules, including numerous neurotransmitter receptors located on the cell membrane, which facilitate signal transduction from the extracellular environment into the cell. In the nervous system, neuroactive ligands bind to specific receptors, initiating a cascade of signal transduction pathways that regulate various neurological functions. The neuroactive ligand–receptor interaction pathway was also significantly enriched by metabolites from cluster 6, one of ten clusters obtained by trend analyses. This cluster included circadian rhythms-related metabolites, such as melatonin, cortisol, and adenosine, indicating the crucial roles that rhythmic metabolites play in the bat brain. Melatonin has properties of antioxidation, anti-inflammation, and antiapoptosis to improve neuroprotective functions [63]. It primarily exerts its effects by binding to membrane-associated G protein-coupled receptors [25]. Studies have shown that melatonin is considered neuroprotective in the nervous system by reducing nitric oxide synthesis or scavenging free radicals, thereby mitigating their harmful effects [64]. Additionally, melatonin’s antioxidant properties effectively reduce oxidative stress during bats’ flight and hunting periods, protecting neural cells from oxidative damage and maintaining the stability of neural activity. Cortisol, one of the most potent hormones in human physiology, targets nearly all cells in the body. It acts as a key mediator in transmitting circadian signals from the suprachiasmatic nucleus (SCN) to peripheral tissues [65]. In addition, cortisol plays a crucial role in energy metabolism and immune response [66]. In bats, during nocturnal activity, cortisol secretion promotes the breakdown of fats and glycogen, providing the large amounts of energy needed for their activities. This energy regulation directly influences the neural activity and flight capabilities of bats.

**Figure 14:**
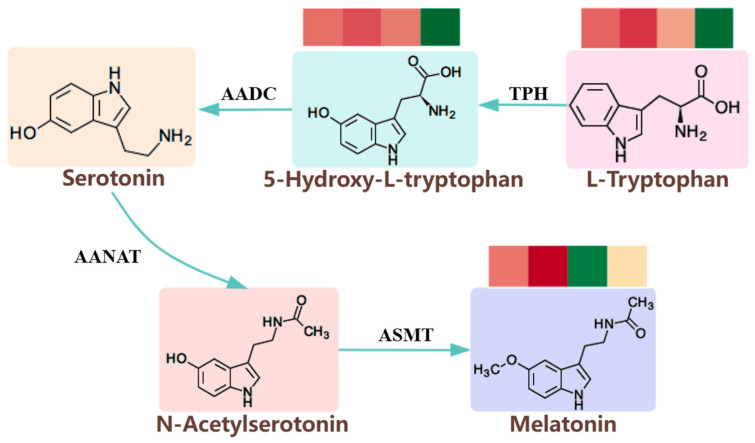
Melatonin synthesis process. The simple heatmap showed the content of corresponding metabolites across four time points from 4:00 to 22:00. The deeper the red color, the higher the content; the deeper the green color, the lower the content.

Notably, melatonin, cortisol, and adenosine all exhibited a daily pattern of highest abundance during sleep (around 10:00 am), followed by a substantial decrease until the wake-up period (around 4:00 pm), and then a progressive increase during active and rest states. Papers have demonstrated that these substances influence rhythmic changes in various aspects of physiological processes, including metabolism, stress responses, and neural activities, particularly in the sleep–wake cycle [67,68]. As widely recognized, melatonin plays a crucial role in regulating circadian rhythms, intrinsic biological timekeepers that govern the sleep–wake cycle. Predominantly synthesized by the pineal gland in response to darkness, melatonin signals the body to prepare for sleep [69]. Melatonin is produced using tryptophan as a precursor, and there are three main pathways of tryptophan metabolism, namely, the kynurenine (Kyn) pathway, serotonin (5-HT) pathway, and indole pathway [70]. In this process, tryptophan is synthesized into melatonin through the serotonin (5-HT) pathway involving a series of enzymes such as N-acetyltransferase [71]. In this study, L-Tryptophan, Melatonin, and the intermediate 5-Hydroxy-L-tryptophan were detected in the process of tryptophan synthesis of melatonin (Figure 14). Their abundance varied in different physiological states, and both exhibited a rhythmic pattern in the bat’s brain. Adenosine acts as a neuromodulator with a general inhibitory effect on neuronal activity [72]. By reducing neuronal activity and inhibiting neurotransmitter release, it promotes sleep and peaking in concentration during bat sleep periods. Moreover, research suggested that the sleep-promoting effects of endogenous melatonin may be mediated by adenosine signaling [73]. For nocturnal animals, cortisol, as a hormone secreted by the adrenal cortex, follows a circadian rhythm, typically peaking in the morning to enhance alertness and energy supply for coping with daytime activities and stress. However, cortisol secretion patterns are influenced by individual lifestyle and environmental factors. In the brain of a bat, we found that cortisol increased gradually from awakening to flight states; however, the exact reasons for the highest levels detected during sleep state remain mostly unclear. It is speculated that this may relate to maintaining organismal vigilance during sleep in the wild or could involve interactions with other internal regulatory mechanisms concerning neural activity, as referred to above.

Melatonin secretion will be restricted during the light phase, elevated at night, and peak in the middle of the night. Recent studies on sheep and humans have indicated that melatonin was released directly into the cerebrospinal fluid (CSF), particularly in the third ventricle [74]. The levels of melatonin in five major brain regions of rats, including the whole brain, pineal gland, and serum samples were measured, and the results demonstrated that the levels of melatonin obtained during the dark period were significantly higher in all tissues compared to the levels obtained during the light period [75]. However, in this study, the level of melatonin in bats’ brains was the highest around 10 a.m., corresponding to the deep sleep period, and lowest around 4:00 p.m., corresponding to the wake period. For nocturnal bats, Asian particolored bats here, melatonin showed higher abundance during light periods than dark periods. Therefore, we reasonably speculate that there may be differences in the melatonin regulatory mechanisms between nocturnal and diurnal animals. For nocturnal mammals, the pattern of melatonin variation may be influenced not only by the light–dark cycle but also by other internal physiological processes, such as neural activity and the sleep–wake cycle. Taken together, the secretion of melatonin is not only related to changes in the light environment but is also regulated by internal complex mechanisms, which need more detailed analyses in the future.

### 3.4. Rhythmic Expression of Core Circadian Clock Genes Per1 and Per2 in the Bat Brain

*Pers* are the essential members of core circadian clock genes that play a pivotal role in regulating circadian rhythms in animals. *Pers* comprises *Per1*, *Per2*, and *Per3*, which are predominantly expressed in both the central and peripheral nervous systems. *Per1* is a core member in circadian rhythms regulating, serving as an input channel for the circadian clock and significantly contributing to daily rhythms of light signal input [76]. Mouse lacking the *Per1* gene exhibit a shortened circadian cycle, impairing their ability to maintain the stability and accuracy of the circadian cycle [77]. *Per2* interacts with neurotransmitters to regulate neurobiological activity in the central nervous system. *Per2* rhythmic expression regulates excitability and circadian rhythms of the hypothalamic–pituitary–adrenal (HPA) axis by integrating light signaling and corticotropin-releasing factor (CRF) stress-related neurotransmitters, leading to circadian rhythms in target organs [78]. Prior studies have revealed rhythmic changes of the *Per1* gene in the brain of Asian particolored bats through comparative brain transcriptomes [23]. In this metabolomics study, correlation analyses between DAMs and DEGs indicated a strong negative correlation between both *Per1* and *Per2* with tryptophan (a precursor to melatonin). This suggested that *Per2* may also play a crucial role in the regulation of circadian rhythm in bats. Research has demonstrated that the regulation mechanism of circadian rhythm within the central nervous system is complex [79,80]. However, only *Per2* can regulate the rhythm periodicity and amplitude of the central and peripheral nervous system [81,82]. *Per2*-immunoreactive (ir) neurons receive neuronal projections from the SCN and the sympathetic nervous system and are regulated by the neuroendocrine system via corticosterone, melatonin, and adrenaline [79,80]. *Per2* mutant mice exhibit a shorter circadian period compared to wild-type (WT) mice, alongside reduced *Per1* expression in the SCN, indicating that *Per2* plays a regulatory role in *Per1* expression [83]. Therefore, we suppose that both *Per1* and *Per2* are involved in circadian rhythm regulation in the bat brain. However, further research is needed to determine whether *Per2* has a unique function in the bat brain and elucidate the related regulatory mechanisms.

## 4. Materials and Methods

### 4.1. Sample Collection

All bats used in this study were collected in July 2022 from Heilongjiang Province, China (45°32′55″ N, 127°32′59″ E). During a week of observation, we recorded and confirmed the daily activities of Asian particolored bats, and selected the same time points as those used in the previous study on the brain transcriptome [23]. From approximately 2:00 a.m. to 6:00 a.m., bats returned successively to their habitats after flying for hunting and engaging sporadically in grooming or emitting calls, which were defined as rest. From 8:00 a.m. to 12:00 p.m., all bats fell asleep. Between approximately 2:00 p.m. and 6:00 p.m., most individuals awakened and engaged in simple activities such as emitting calls and fluttering. From approximately 8:00 p.m. to 12:00 p.m., most bats flew out for hunting, defined as an active period. Accordingly, four respective sampling time points corresponding to four different states of bats were confirmed: 4:00 a.m. (rest), 10:00 a.m. (sleep), 4:00 p.m. (wake), and 10:00 p.m. (activity). For the active state, bats were captured by mist nets, and for the states of rest, sleep, and wake, bats were captured by hand-draft nests. Six biological replicates were included for each time point, and 24 adult individuals were collected in total, with weights averaging around 20 g. Most individuals in the bat colony were female and, to avoid the influence of sex-related differences, only females were selected for inclusion in this study. The bats were sacrificed by decapitation at the four time points during the day to minimize suffering. Brain tissues from each individual were collected and flash-frozen in liquid nitrogen as quickly as possible followed by storage in a −80 °C freezer until further processing for total metabolite extraction [84,85].

### 4.2. Metabolite Extraction and Sequencing Sample Preparation

Each bat brain tissue was ground into powder separately, mixed evenly, and then equal amounts of brain samples (25 mg, n = 24) were weighed. Homogenate beads mixed with 500 μL of extraction solution (methanol:acetonitrile:water = 2:2:1) were added. The samples were homogenized at 35 Hz for 4 min and sonicated for 5 min in an ice–water bath. This homogenization and sonication cycle was repeated three times. Afterward, the samples were centrifuged at 12,000 rpm (RCF = 13,800× *g*, R = 8.6 cm) for 15 min at 4 °C, and the supernatant was transferred into a detection vial. Meanwhile, quality control (QC) samples were prepared by pooling extracts from different sample groups and processed and tested in the same way as the experimental samples. All samples were stored at −80 °C prior to LC-MS analysis. The following are details of the reagents used in this part, regent name, CAS, and brand, respectively: Methanol, 67-56-1, CNW Technologies, Düsseldorf, Germany; Acetonitrile, 75-05-8, CNW Technologies; ddH_2_O, Sangon Biotech, Shanghai, China.

### 4.3. LC-MS-Based Metabolomic Analysis

All samples were analyzed using an Ultra-High-Performance Liquid Chromatography (Vanquish, Thermo Fisher Scientific, Waltham, MA, USA) coupled to Q Exactive HFX mass spectrometer (Orbitrap MS, Thermo Fisher Scientific, Waltham, MA, USA) to perform metabolic analysis in positive and negative ion modes. UHPLC chromatographic separation was carried out on an Acquity UHPLC system (Acquity LC, Waters, Milford, MA, USA) equipped with a Waters UPLC column (ACQUITY UPLC BEH Amide 1.7 µm, 2.1 mm × 100 mm, Waters, Milford, MA, USA). The mobile phase A consisted of 25 mM ammonium acetate and 25 mM ammonium hydroxide in water, while mobile phase B was 100% ACN. The flow rate was set to 0.5 mL/min, with an injection volume of 2 µL, and samples were maintained at 4 °C in the autosampler. The QE HFX mass spectrometer acquired MS/MS spectra in information-dependent acquisition (IDA) mode, controlled by Xcalibur software version 4 (Thermo Fisher Scientific, Waltham, MA, USA). In this mode, the software continuously evaluates the full scan MS spectrum. The ESI source conditions were as follows: sheath gas flow rate at 50 Arb, auxiliary gas flow rate at 15 Arb, capillary temperature 320 °C, full MS resolution as 60,000, MS/MS resolution at 15,000, collision energy set at SNCE 20/30/40, and spray voltage at 3.8 kV (positive) or −3.4 kV (negative). Quality control (QC) samples, created by pooling all samples, were injected at regular intervals throughout the analytical run to assess repeatability. The following are details of the reagents used in this part of the method, regent name, CAS, and brand, respectively: Ammonium acetate, 631-61-8, Sigma-Aldrich, St. Louis, MO, USA; Ammonium hydroxide, 1336-21-6, Fisher Chemical, Pittsburgh, PA, USA; ddH_2_O, Sangon Biotech.

### 4.4. Metabolomic Data Processing

The raw data were converted to the mzXML format using ProteoWizard version 3.0 and processed with an in-house program, which was developed using R and based on XCMS, for peak detection, extraction, alignment, and integration. The R package and the AllwegeneDB were applied in metabolite identification. Compound identification was based on the precise mass-to-charge ratio (*m*/*z*), secondary fragments, and isotopic distribution using the Human Metabolome Database (HMDB, http://www.hmdb.ca, accessed on 5 January 2023), The Kyoto Encyclopedia of Genes and Genomes (KEGG, http://www.kegg.jp, accessed on 6 January 2023), Metlin, CAS, Pubchem, and chEBL retrieval databases to perform qualitative analysis. The extracted data were then further processed by removing any peaks with a missing value (ion intensity = 0) in more than 50% of groups, and by screening according to the qualitative results of the compound. Even after filtering data for missing values up to 50%, some missing values may remain due to various reasons contributing to differential frequencies of missing data across variables. Such discrepancies can skew group distributions and variances, potentially leading to inaccurate statistical analyses and data interpretations. Therefore, the missing values in the raw data were filled with half of the minimum value [86]. Additionally, an internal standard normalization method was employed in this data analysis. Features with RSD >30% were excluded from the further analysis. The resulting three-dimensional data, including peak number, sample name, and normalized peak area, were integrated and imported into R (v4.2.3) for principal component analysis (PCA) and orthogonal partial least squares discriminant analysis (OPLS-DA). PCA was used to visualize the overall metabolic differences between groups and assess the variability among samples within the same group [87], helping to evaluate intra-group similarity and inter-group differences and eliminate outliers. To achieve better group separation and identify variables responsible for classification, supervised OPLS-DA was applied to calculate the R2 and Q2 values. R2 indicates how well the variation in a variable is explained, while Q2 reflects how well a variable can be predicted. To prevent overfitting, sevenfold cross-validation and 200-response permutation testing (RPT) were used to assess model quality. Variable importance of projection (VIP) values obtained from the OPLS-DA model were used to rank the overall contribution of each variable to group discrimination.

### 4.5. Differentially Accumulated Metabolites (DAMs) Identified

To clarify the differences in the metabolic levels in the bat brain over a 24 h period, metabolites at two adjacent time points were compared and analyzed sequentially: rest vs. sleep, sleep vs. wake, wake vs. activity, and activity vs. rest. Additionally, two groups of the exact opposite states were compared, rest vs. wake and sleep vs. activity. A total of six pairwise comparisons were conducted, consistent with previous transcriptomic studies of the bat brain [23]. The criteria for identifying DAMs in each comparison group were VIP > 1, Fold Change > 1.5 or < 2/3, and *p*-value < 0.05. Based on the KEGG database, metabolic pathway enrichment analysis was performed for the DAMs from six pairwise comparisons, and the hypergeometric test was used to identify significant enrichment pathways (adjusted *p*-value < 0.05).

### 4.6. Rhythmic Analysis of Metabolites

To better uncover the rhythmic metabolites in the bat brain, rhythmic analyses were conducted on all metabolites as well as all DAMs (detected from six pairwise comparisons), respectively. The Cosinor and JTK_CYCLE methods, embedded in DiscoRhyth, a framework for analyzing the periodicity of large-scale temporal biological datasets, were used to identify metabolites with statistically significant rhythmic properties (*p* < 0.05). Only metabolites detected by both algorithms were strictly considered rhythmic.

### 4.7. WGCNA Visualization of Metabolite Networks

To identify the metabolite sets significantly associated with each state across all metabolites, and to reveal the key metabolites and pathways for each state, we used the WGCNA package in the R software (v4.2.3) to analyze the correlation between all brain metabolites and the four physiological states. The results obtained from WGCNA were then imported into Cytoscape software (v3.9.1) to generate a metabolite interaction network. Key metabolites were identified using a topological network algorithm in the Cytohubba plug-in of Cytoscape software. Subsequently, KEGG functional enrichment analysis was performed for the metabolites from the target modules that showed a significant correlation with specific physiological states.

### 4.8. Correlation Analysis between Metabolomics and Transcriptomics

We undertook a search for common pathways as follows: the pathways that were significantly enriched by DAMs and DEGs from the same pairwise comparisons were analyzed to determine the physiological processes in which metabolites and genes jointly participate in regulation.

Correlation analysis of DAMs and DEGs was as follows: The Pearson correlation method was used to calculate the correlation between DAMs and DEGs. Metabolites and genes with a correlation coefficient > 0.8 and a *p*-value < 0.05 were selected, and their expression patterns were analyzed using a nine-quadrant diagram.

## 5. Conclusions

For the first time, this study conducted metabolomics of the whole brain in bats, revealing the variation patterns of key rhythmic metabolites, such as melatonin and tryptophan. Various rhythmic physiological processes involving rhythmic metabolites were identified in the bat brain, including energy metabolism, signaling molecule interactions, and endocrine hormone regulation. Notably, the pentose phosphate pathway was found to play a particularly significant role before aerial predation, and we highlighted its importance in coordinating with oxidative phosphorylation to supply sufficient energy and raw materials to support the high-intensity flight of bats. In future research, with advancements in science and technology, there is the potential opportunity to conduct partitioned detection of the brains of small-sized animals like bats, allowing for a deeper understanding of circadian clock regulation in their brains. At the molecular level, single-cell transcriptomics could be utilized to reveal the functional differences between cell types in circadian rhythm regulation. In conclusion, this study provides insights into the metabolic characteristics of bats at different time points of the day, deepens our understanding of the molecular and physiological bases underlying circadian rhythms in nocturnal bats, and provides valuable references for future studies on rhythmic processes in non-model animals.

## Figures and Tables

**Table 1:** The number of DAMs identified from six pairwise comparisons and the number of pathways significantly enriched by DAMs.

Comparable Group	Up DAMs	KEGG Pathway		Down DAMs	KEGG Pathway		Total DAMs
		*p* < 0.05	Adjusted *p* < 0.05		*p* < 0.05	Adjusted *p* < 0.05	
Rest vs. Sleep	48	6	0	62	13	0	110
Sleep vs. Wake	28	10	0	20	11	0	48
Wake vs. Activity	71	20	3	30	8	1	101
Activity vs. Rest	69	6	0	96	19	5	165
Rest vs. Wake	42	5	0	62	9	0	104
Sleep vs. Activity	79	18	3	32	2	0	111

Notes: In terms of the definition of an upregulated quantity of a particular metabolite in each paired comparison, upregulated quantity means that the abundance of the metabolite in the previous time point is higher than the abundance in the later time point, and vice versa.

## Data Availability

The original contributions presented in this study are included in the Supplementary Materials; further inquiries can be directed to the corresponding authors.

## Supplementary Materials

The supporting information can be downloaded at: https://www.mdpi.com/article/10.3390/ijms25189850/s1.
