# Supplementary figures and images for: Daily Brain Metabolic Rhythms of Wild Nocturnal Bats
Source: Int J Mol Sci. 2024 Sep 12;25(18):9850. doi: 10.3390/ijms25189850 (PMC11432702; doi:10.3390/ijms25189850)

# Scores Plot

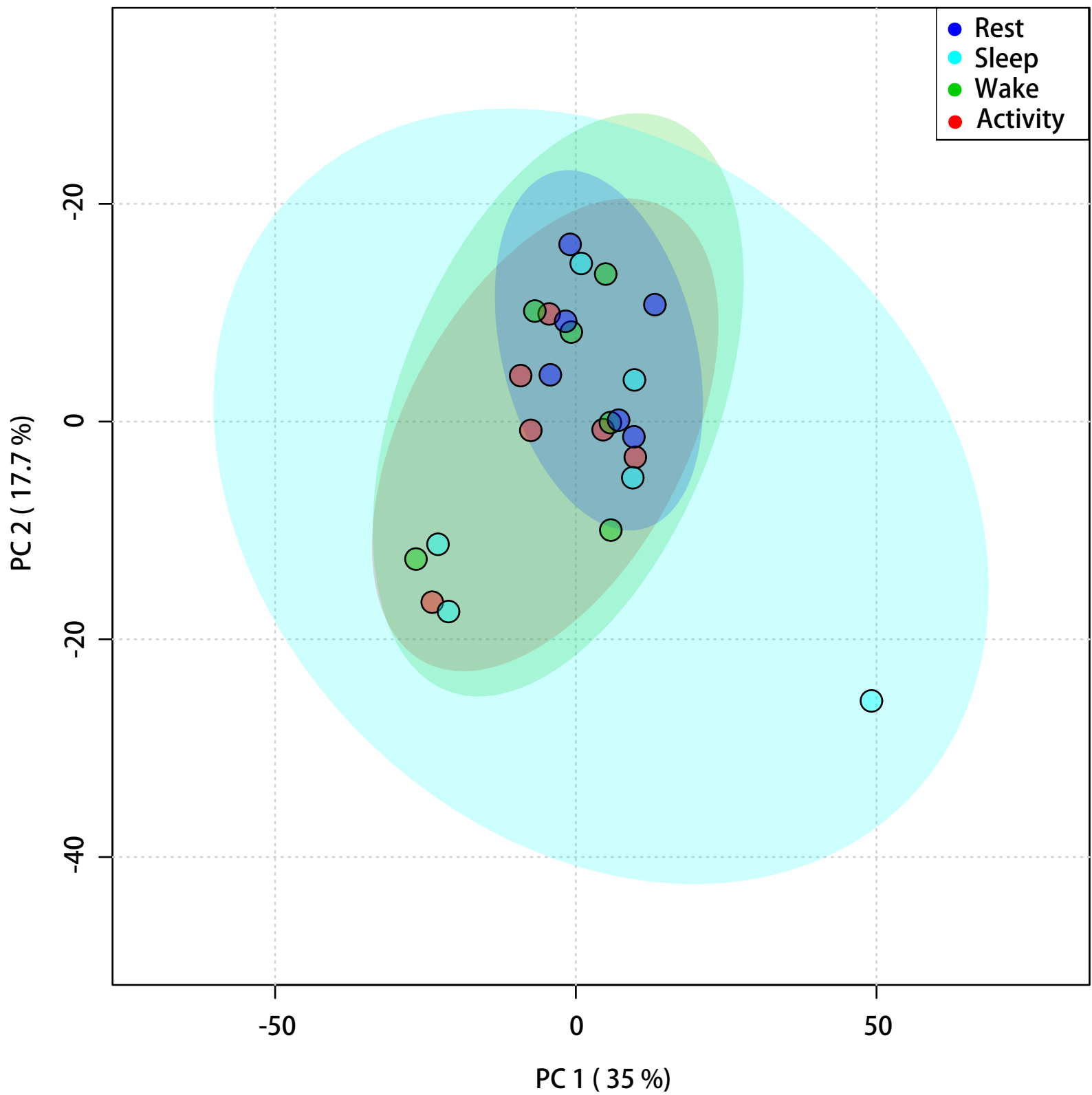

Supplement: Supplementary file 1 [file ijms-25-09850-s001.zip › Figure S1.pdf]

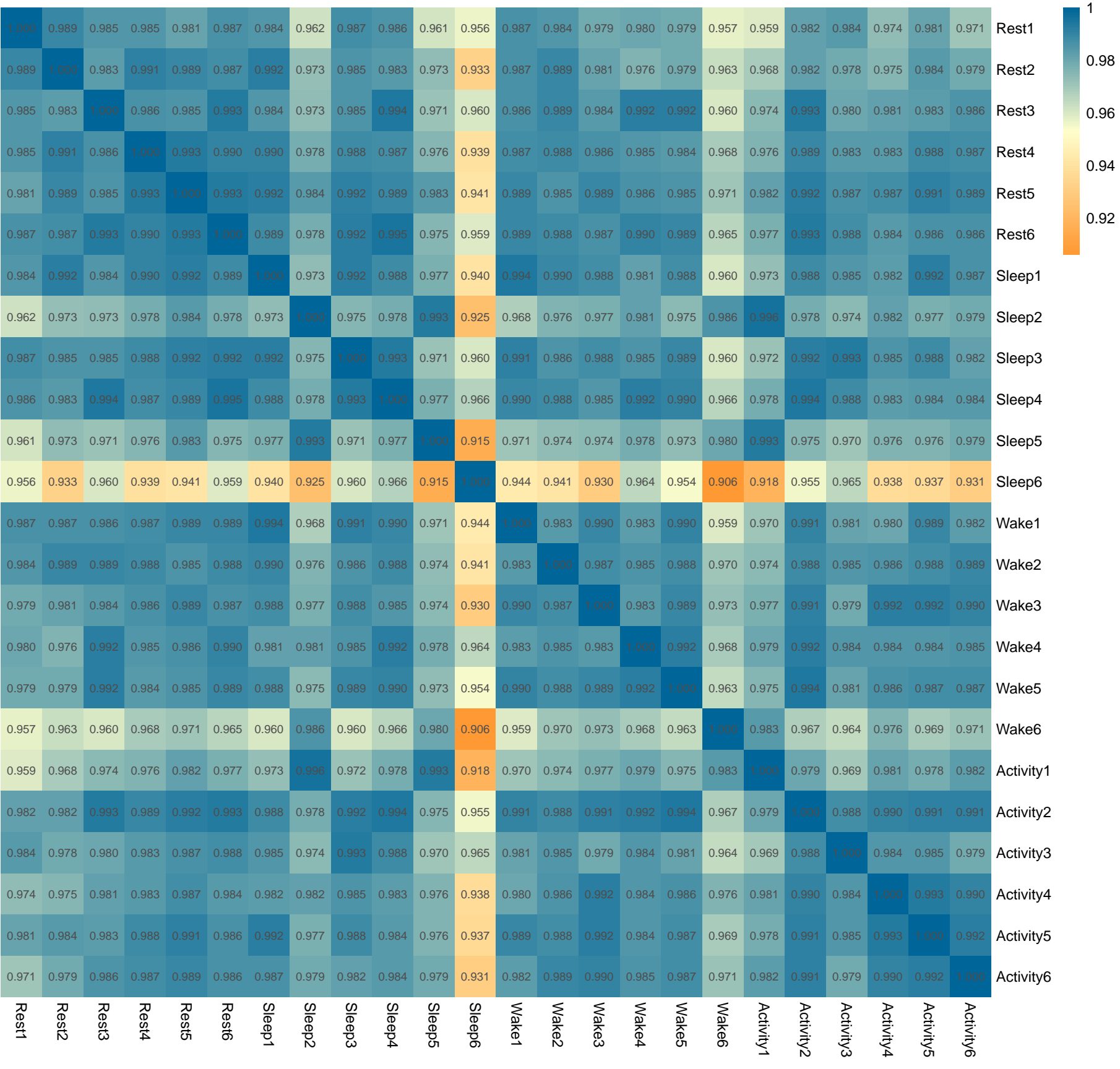

Supplement: Supplementary file 1 [file ijms-25-09850-s001.zip › Figure S2.pdf]

Rest vs Sleep

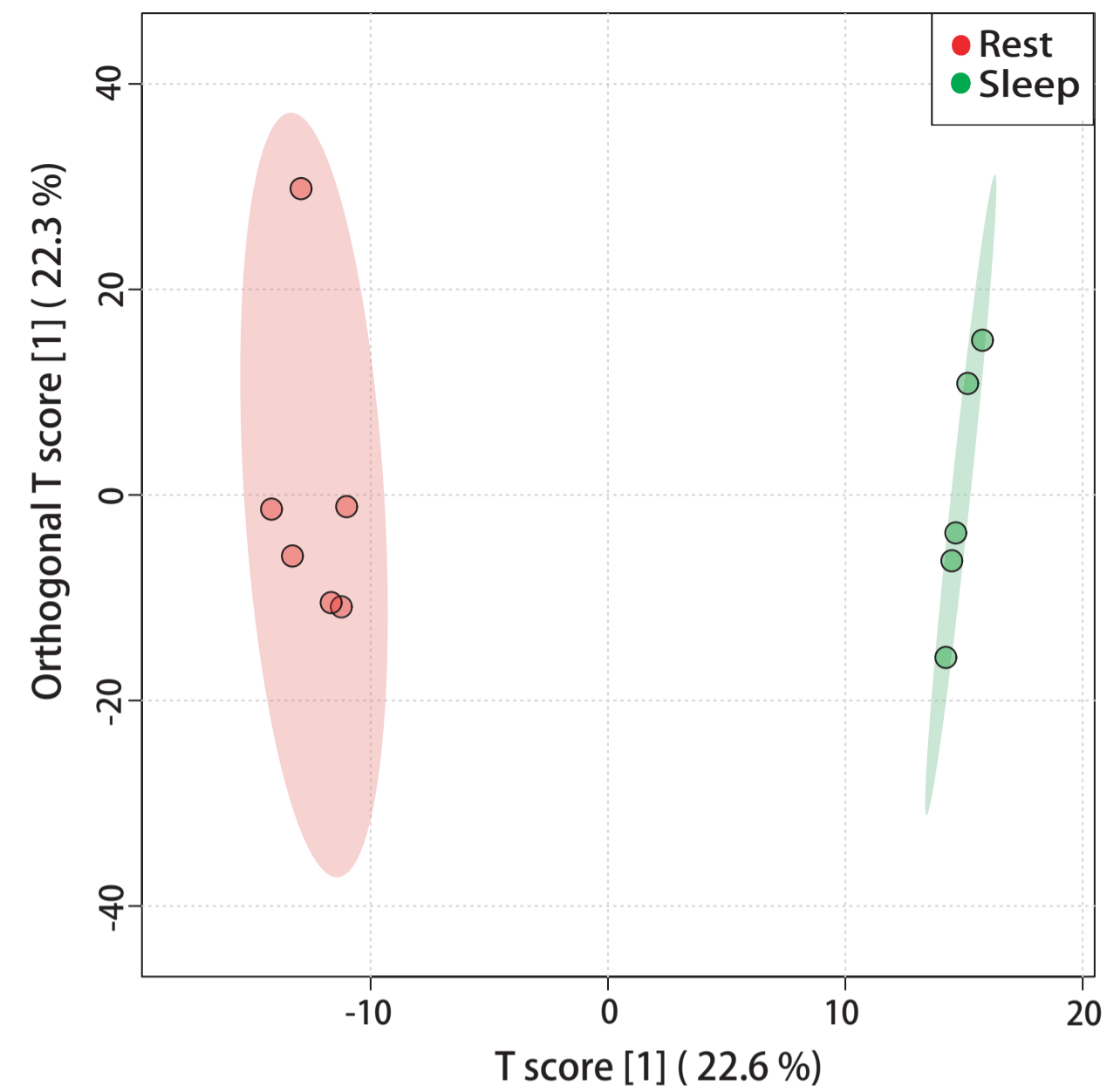

Sleep vs Wake

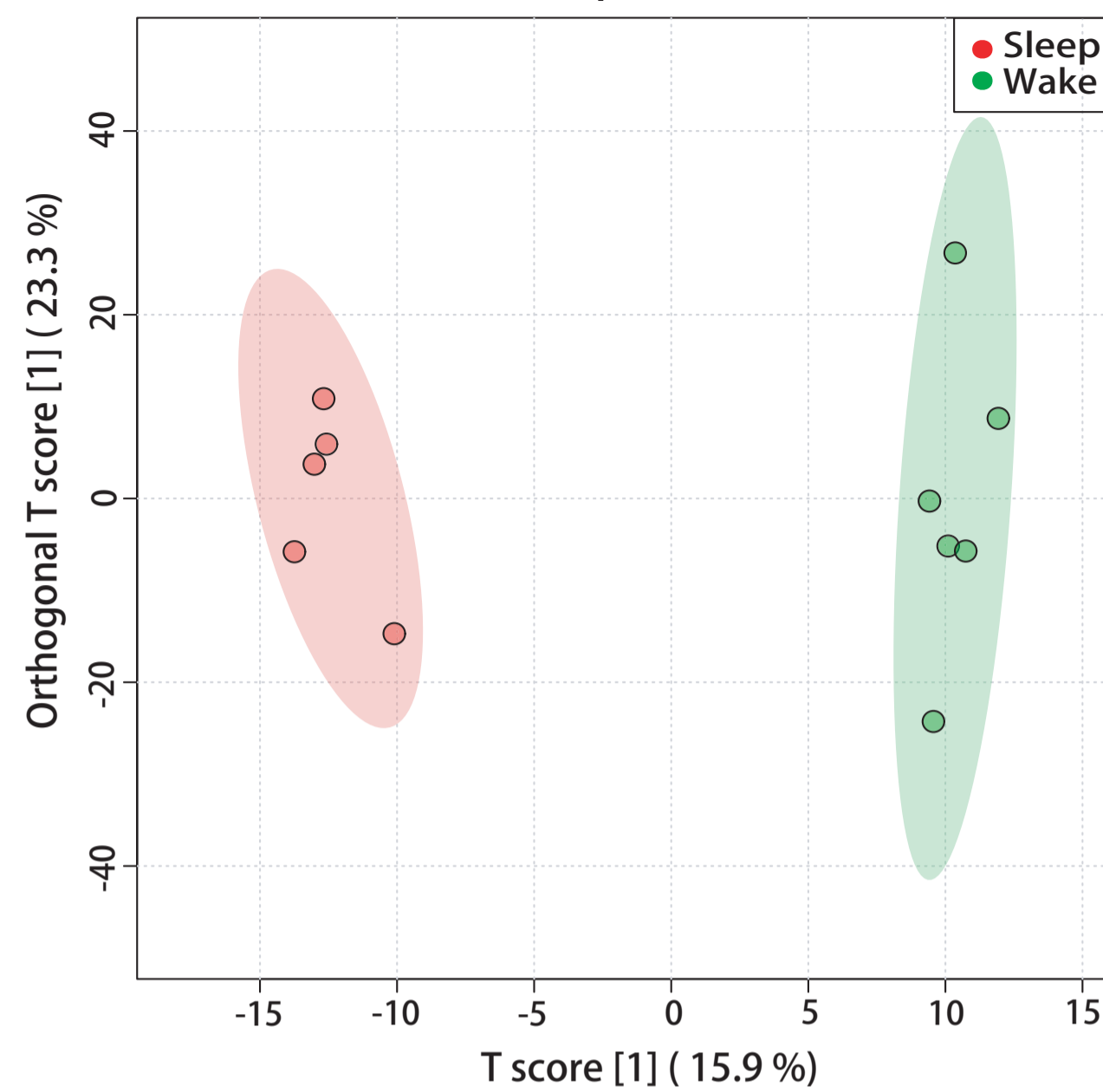

Wake vs Activity

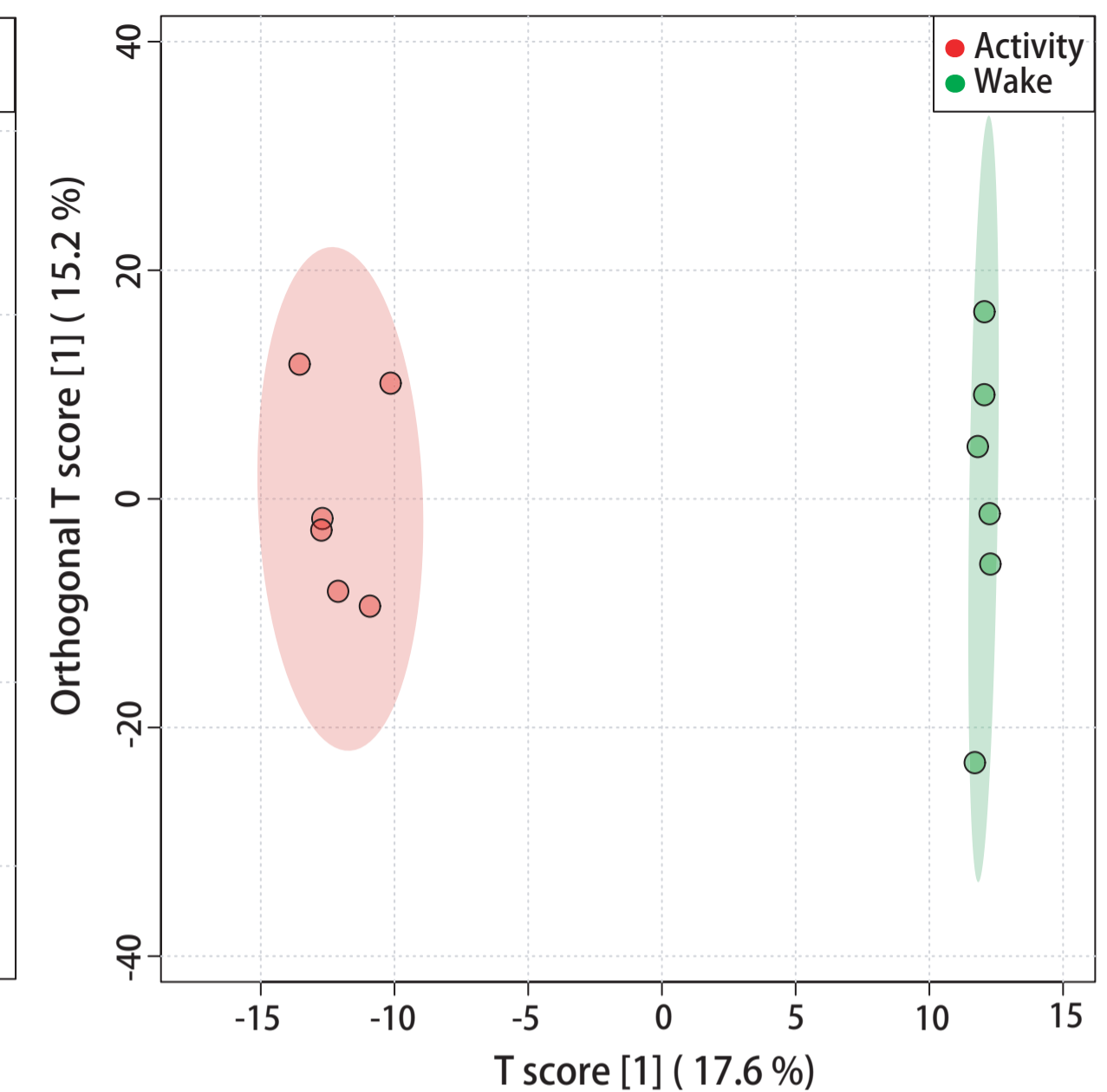

Activity vs Rest

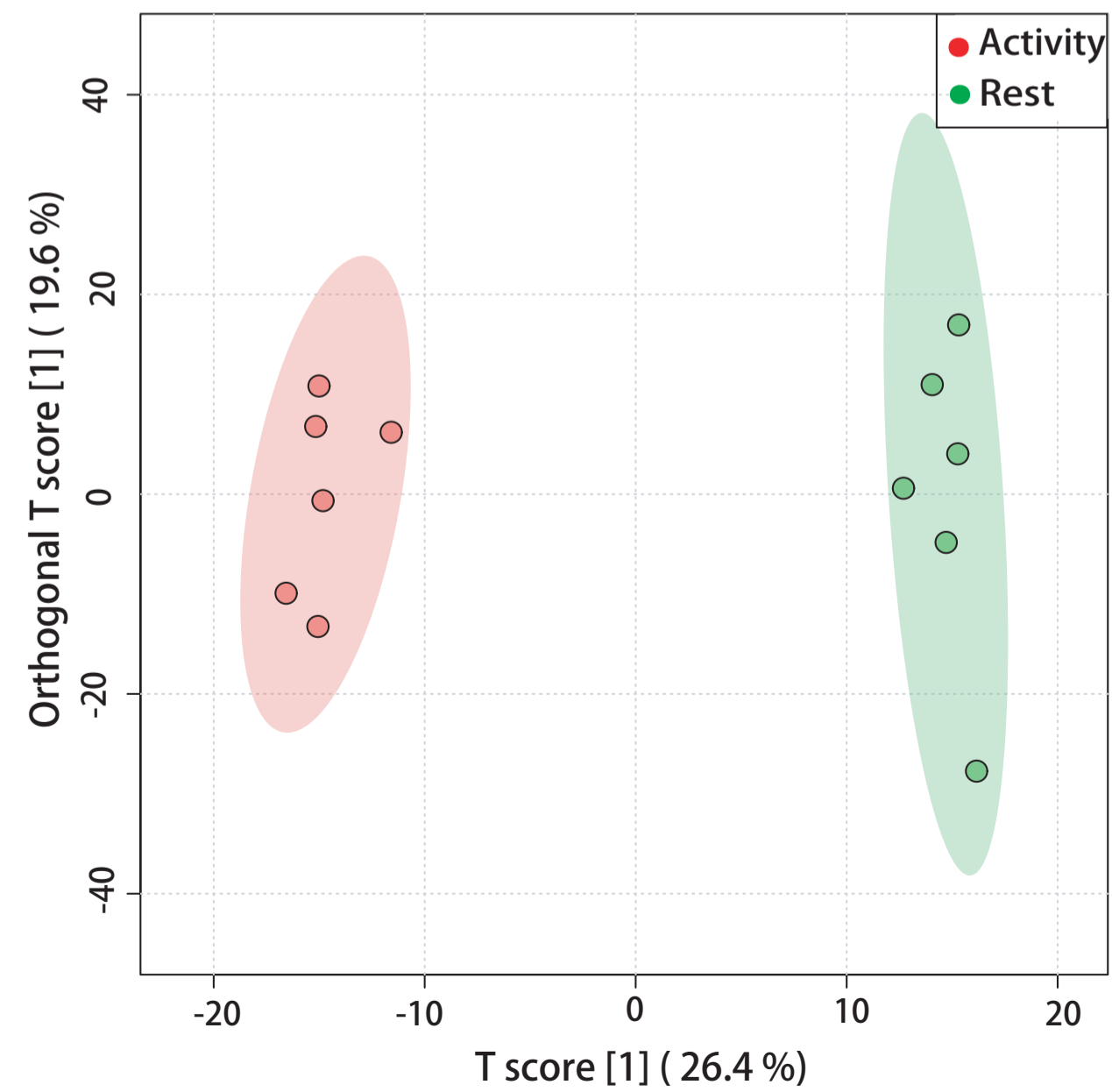

Rest vs Wake

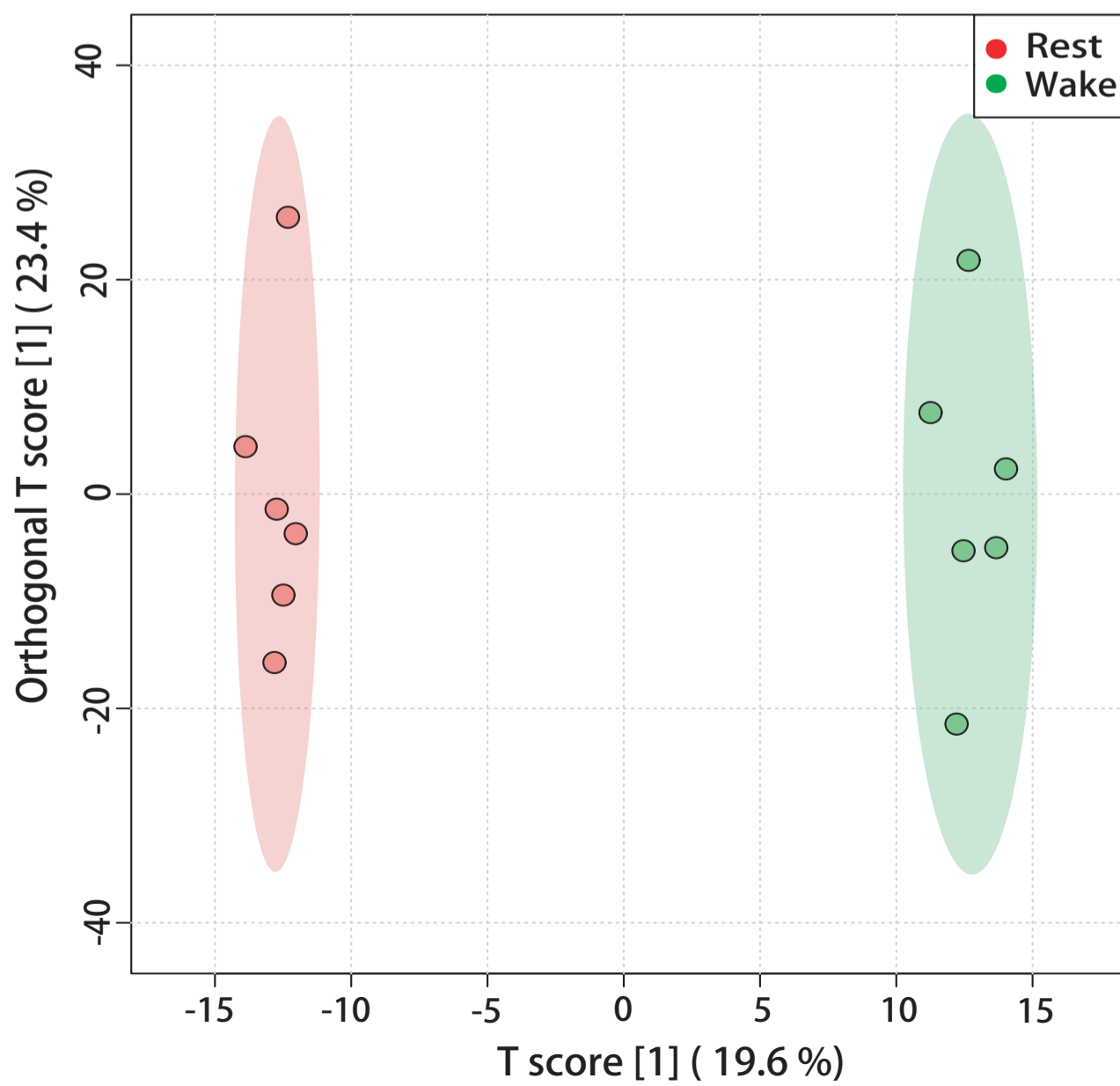

Sleep vs Activity

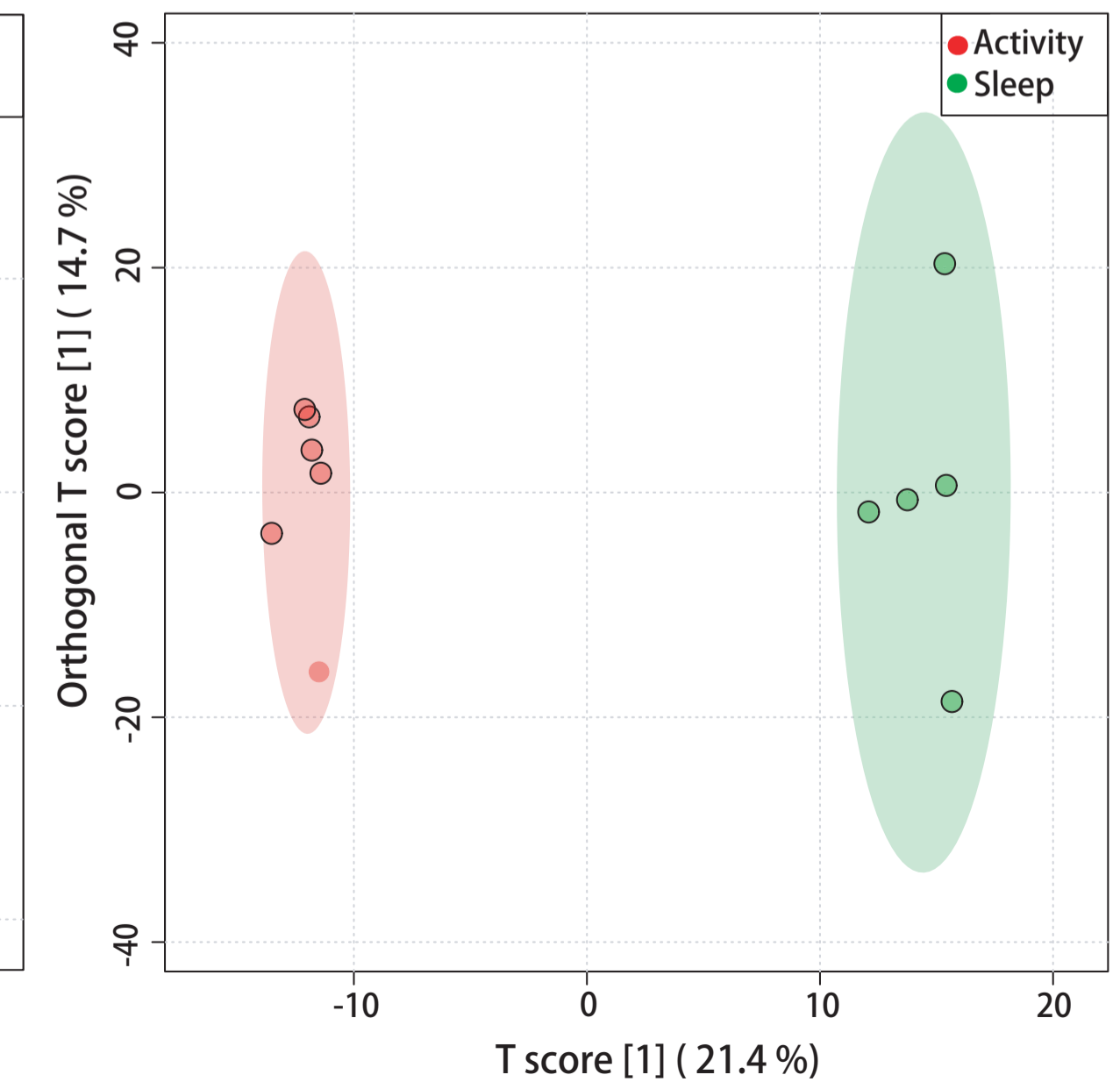

Supplement: Supplementary file 1 [file ijms-25-09850-s001.zip › Figure S3.pdf]

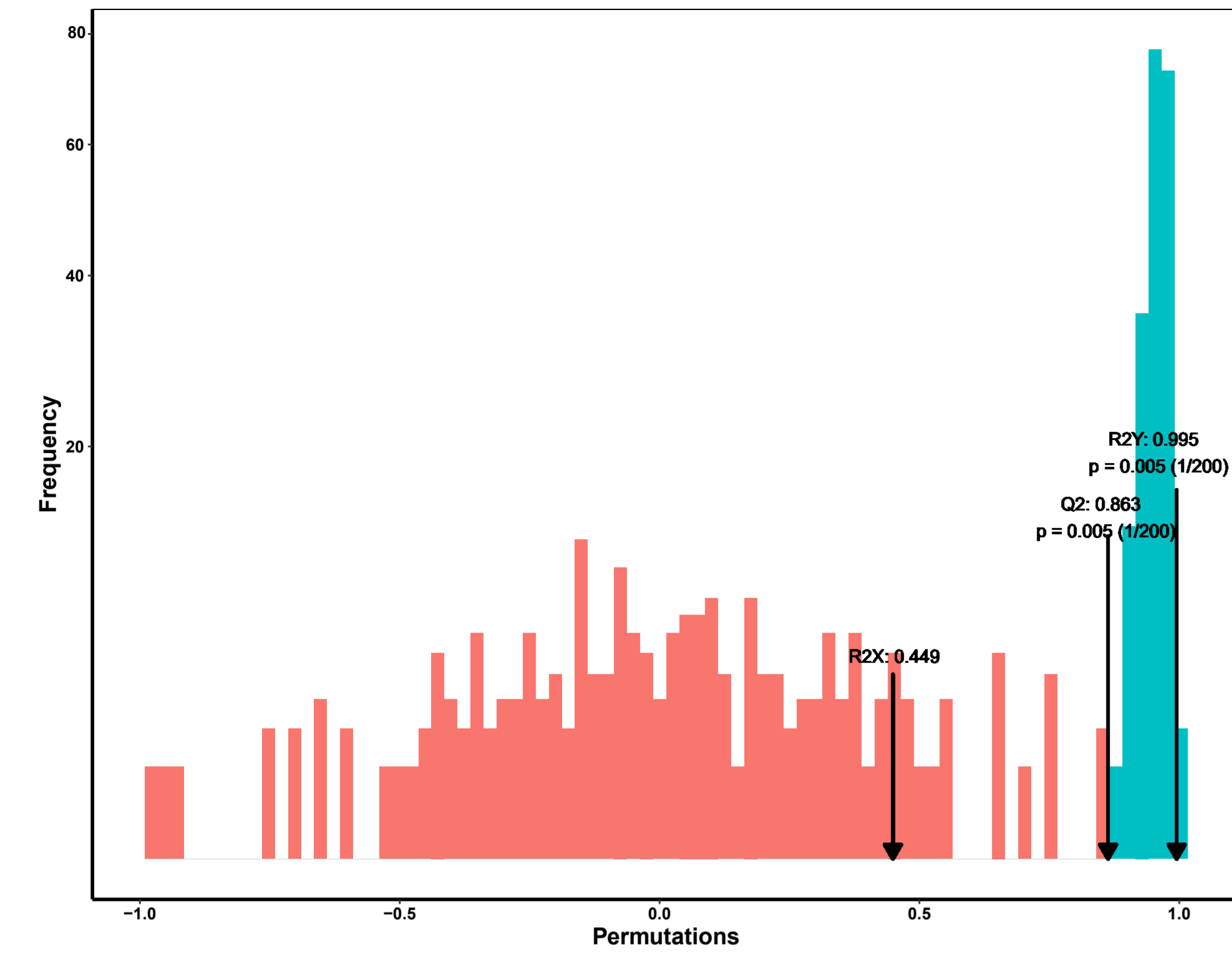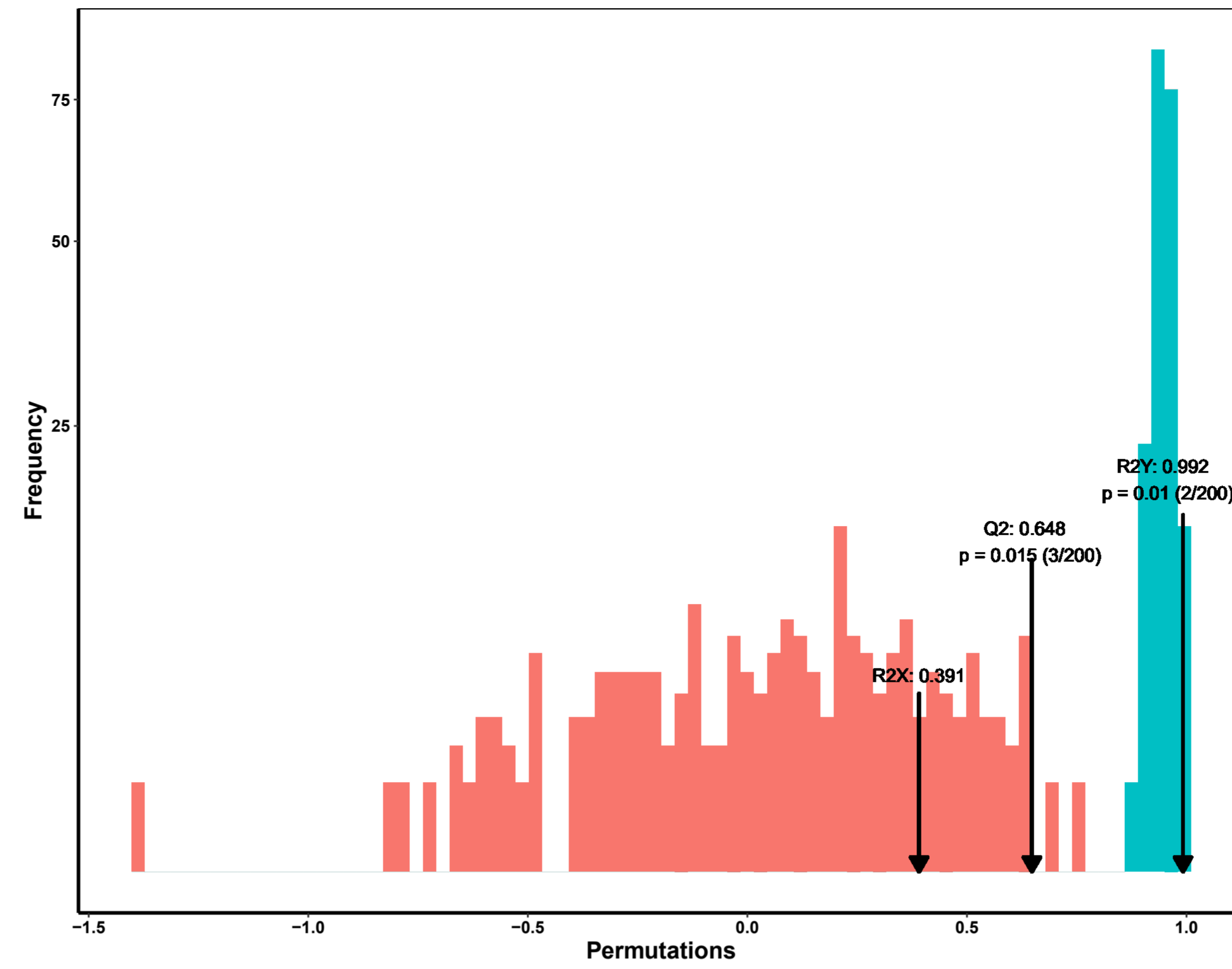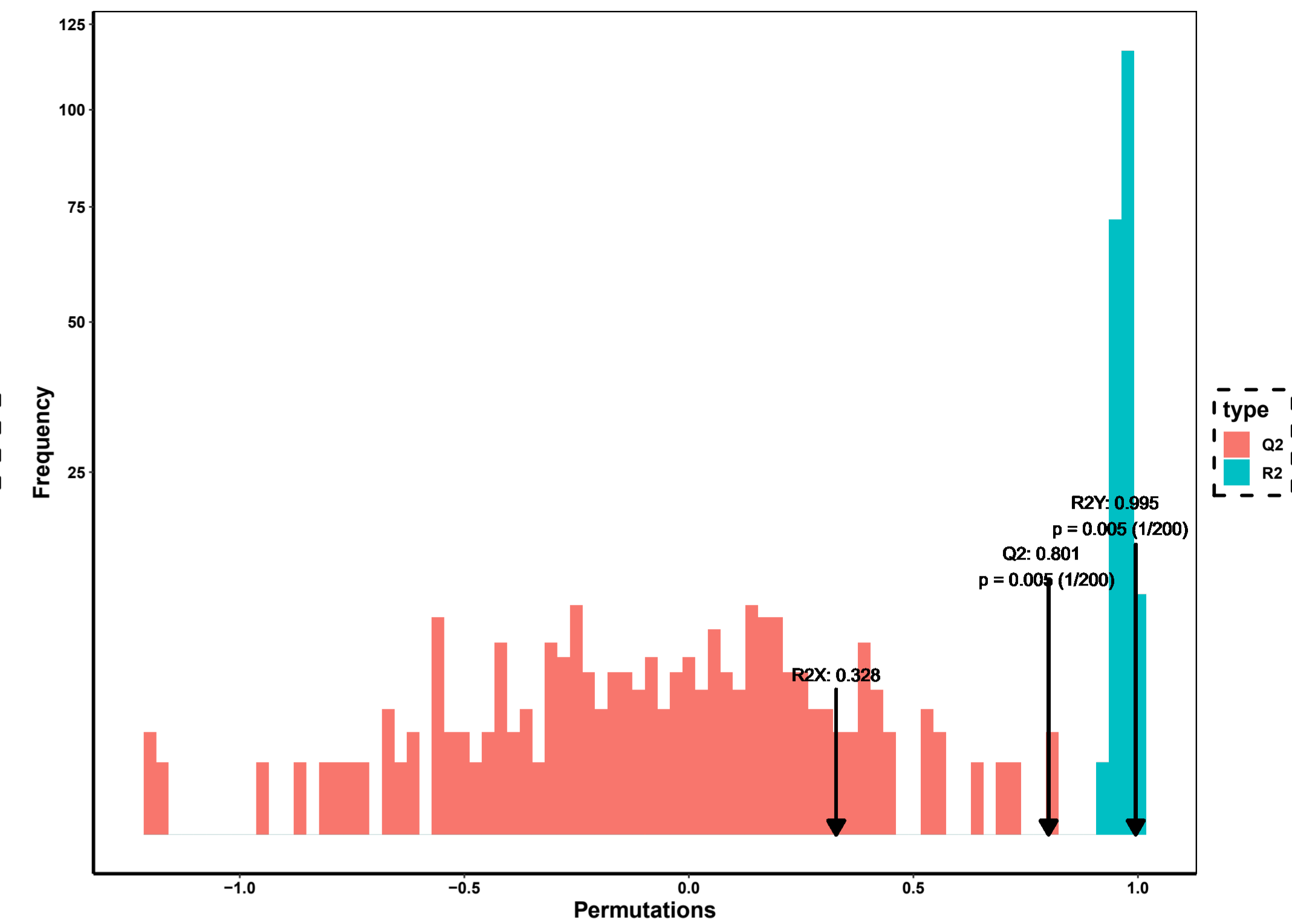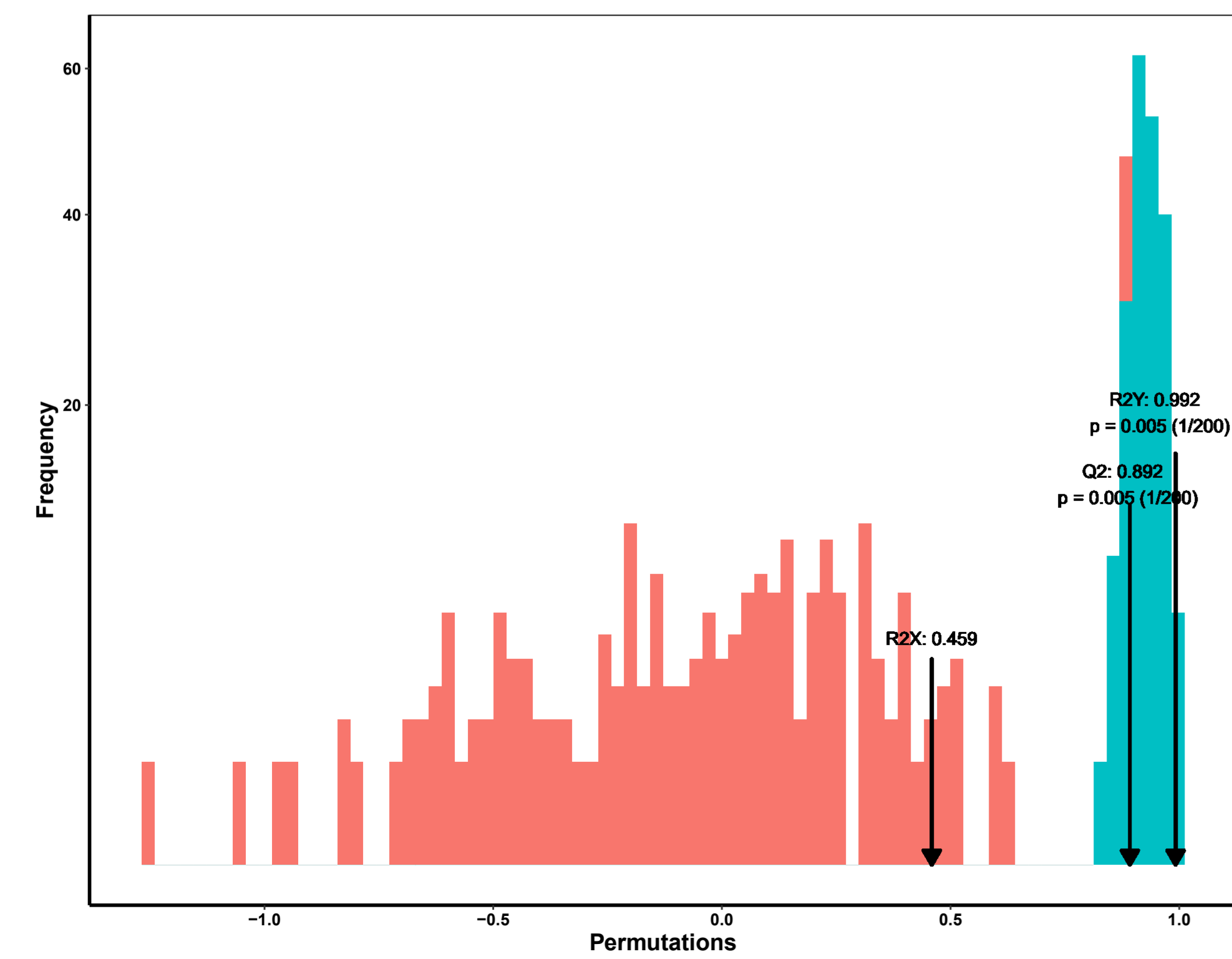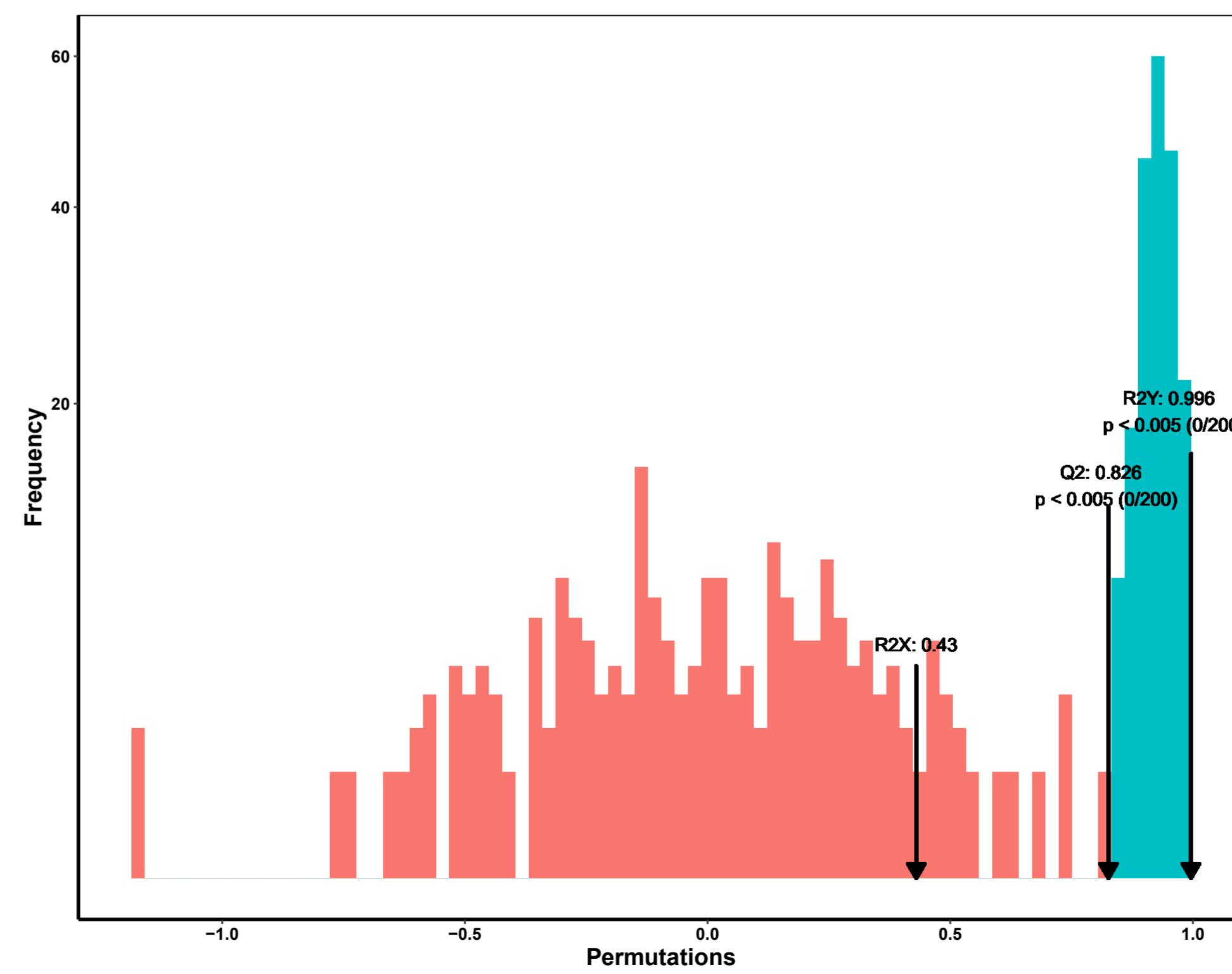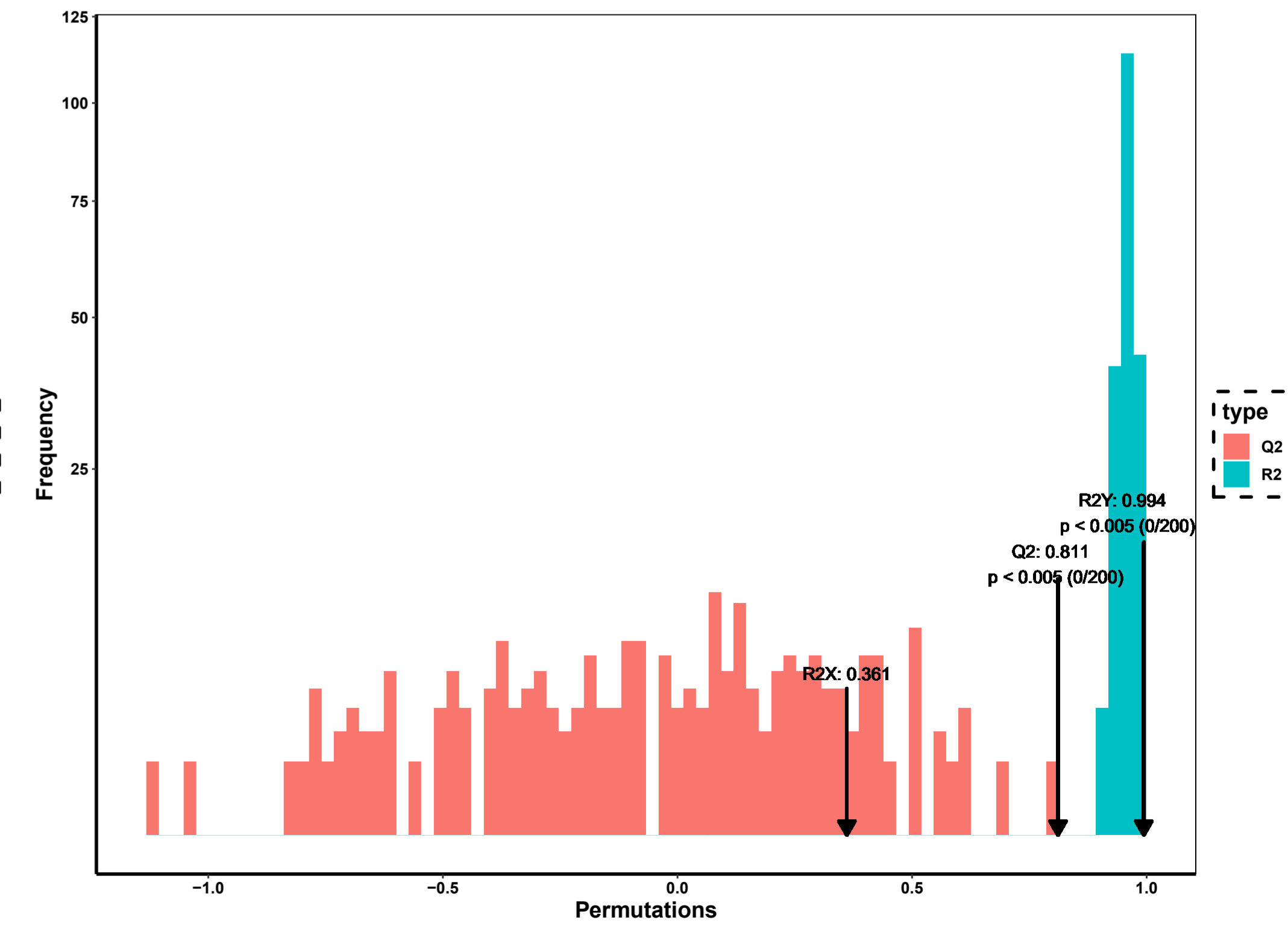

Supplement: Supplementary file 1 [file ijms-25-09850-s001.zip › Figure S4.pdf]

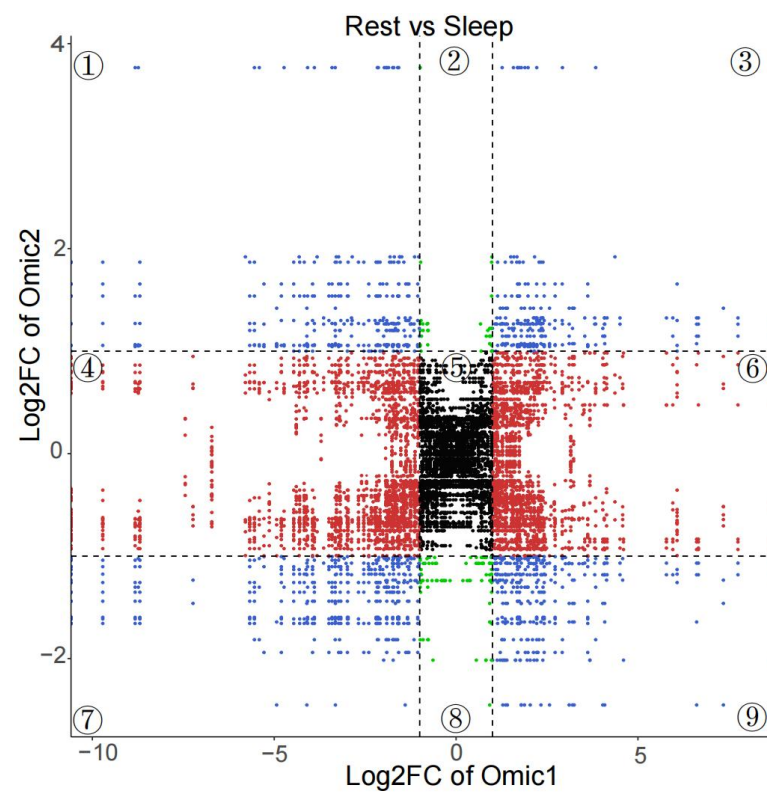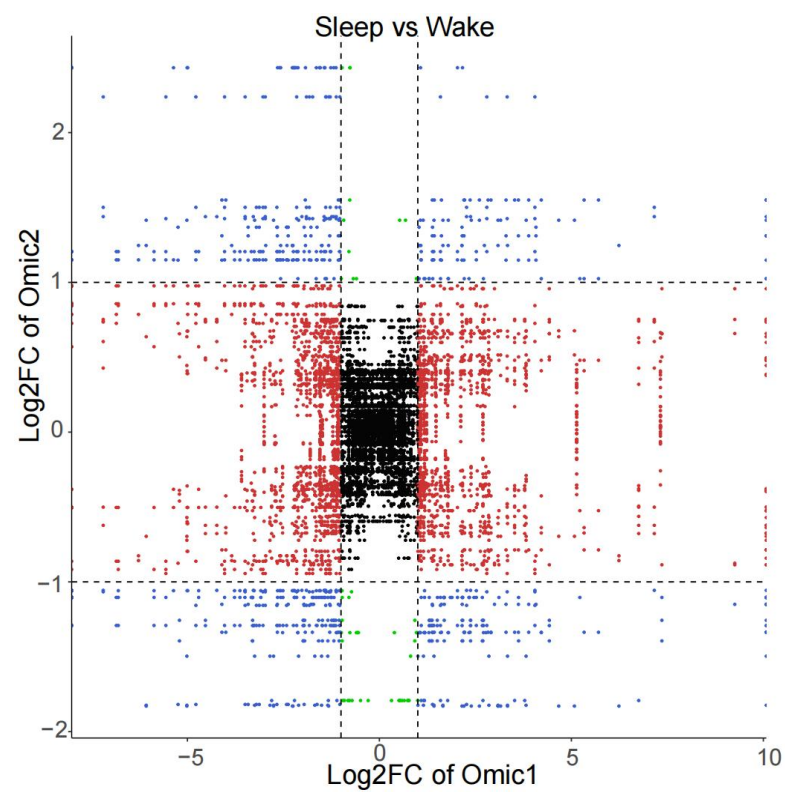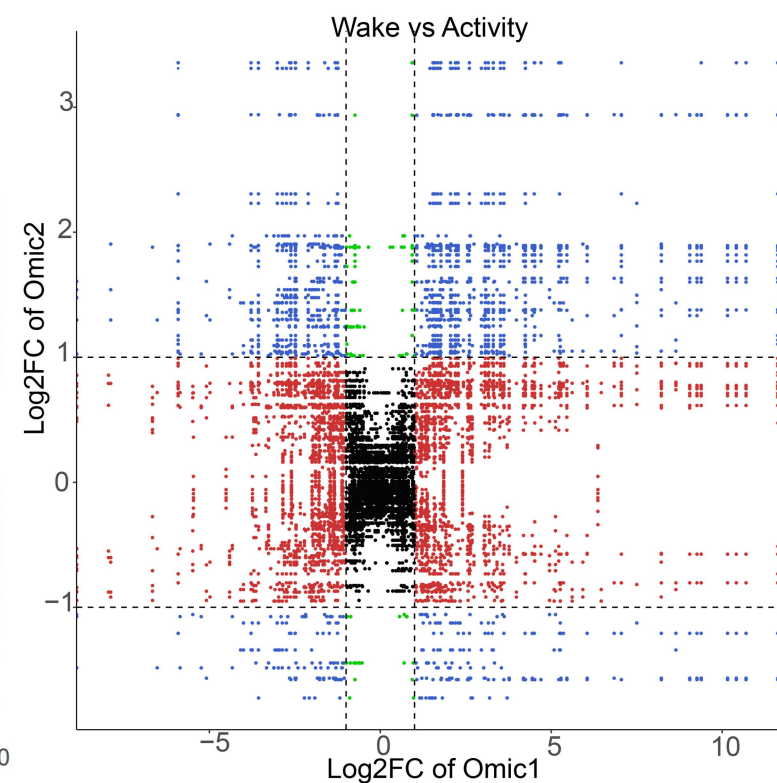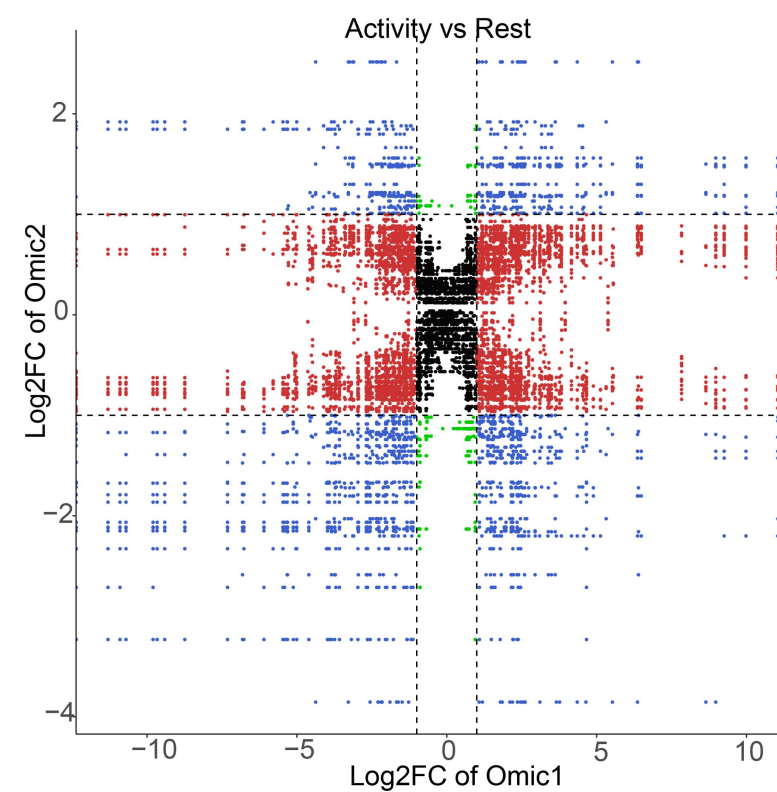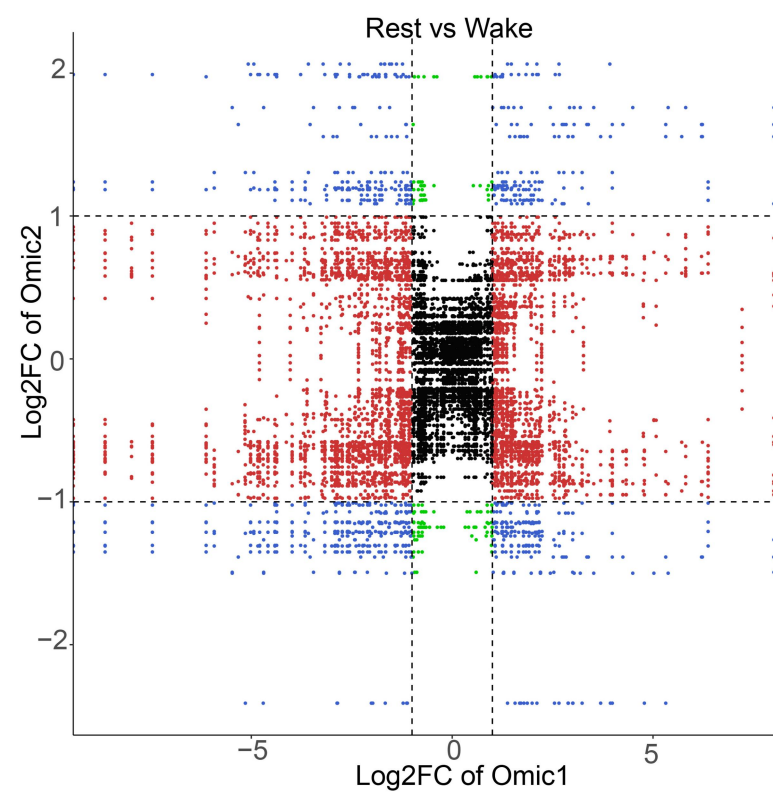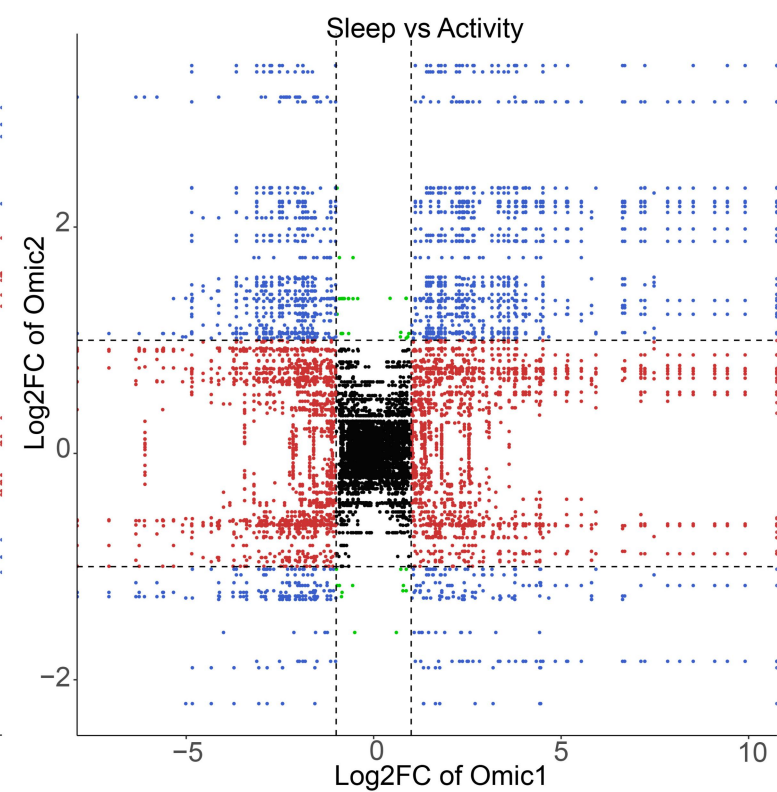

Supplement: Supplementary file 1 [file ijms-25-09850-s001.zip › Figure S5.pdf]
